# Reducing micronutrient deficiencies in Pakistani children: are subsidies on fortified complementary foods cost-effective?

**DOI:** 10.1017/S1368980018001660

**Published:** 2018-07-18

**Authors:** Simon Wieser, Beatrice Brunner, Christina Tzogiou, Rafael Plessow, Michael B Zimmermann, Jessica Farebrother, Sajid Soofi, Zaid Bhatti, Imran Ahmed, Zulfiqar A Bhutta

**Affiliations:** 1 Winterthur Institute of Health Economics, Zurich University of Applied Sciences, Winterthur, Switzerland; 2 Institute of Food, Nutrition and Health, Swiss Federal Institute of Technology Zurich, Zurich, Switzerland; 3 Department of Paediatrics and Child Health, The Aga Khan University, 74800 Karachi, Pakistan; 4 Robert Harding Chair in Global Child Health & Policy, The Hospital for Sick Children, Toronto, Ontario, Canada

**Keywords:** Pakistan, Malnutrition, Micronutrient, Children, Cost-effective, Complementary food, Subsidy

## Abstract

**Objective:**

To estimate the cost-effectiveness of price subsidies on fortified packaged complementary foods (FPCF) in reducing iodine deficiency, iron-deficiency anaemia and vitamin A deficiency in Pakistani children.

**Design:**

The study proceeded in three steps: (i) we determined the current lifetime costs of the three micronutrient deficiencies with a health economic model; (ii) we assessed the price sensitivity of demand for FPCF with a market survey in two Pakistani districts; (iii) we combined the findings of the first two steps with the results of a systematic review on the effectiveness of FPCF in reducing micronutrient deficiencies. The cost-effectiveness was estimated by comparing the net social cost of price subsidies with the disability-adjusted life years (DALY) averted.

**Setting:**

Districts of Faisalabad and Hyderabad in Pakistan.

**Subjects:**

Households with 6–23-month-old children stratified by socio-economic strata.

**Results:**

The lifetime social costs of iodine deficiency, iron-deficiency anaemia and vitamin A deficiency in 6–23-month-old children amounted to production losses of $US 209 million and 175 000 DALY. Poor households incurred the highest costs, yet even wealthier households suffered substantial losses. Wealthier households were more likely to buy FPCF. The net cost per DALY of the interventions ranged from a return per DALY averted of $US 783 to $US 65. Interventions targeted at poorer households were most cost-effective.

**Conclusions:**

Price subsidies on FPCF might be a cost-effective way to reduce the societal costs of micronutrient deficiencies in 6–23-month-old children in Pakistan. Interventions targeting poorer households are especially cost-effective.

Child undernutrition is endemic in Pakistan, with nearly half of children under 5 years of age being stunted and one in three being underweight^(^ ^1^ ^,^ ^2^ ^)^. Undernutrition and micronutrient deficiencies (MND) have severe health consequences and are particularly harmful in early childhood. They irreversibly impair children’s physical and cognitive development^(^ ^3^ ^–^ ^5^ ^)^ and increase mortality risk^(^ ^6^ ^)^.

UNICEF’s infant and young child feeding guidelines^(^ ^7^ ^)^ aim to maximize exclusive – and later to continue partial – breast feeding, with the introduction of appropriate complementary feeding after the sixth month. Complementary feeding is important, as children in this age group undergo rapid mental and physical development with a particularly high need for micronutrient intake. Their micronutrient stores are slowly depleted, yet their diet provides insufficient micronutrients^(^ ^8^ ^,^ ^9^ ^)^. Even in rich countries, sufficient micronutrient intake by 6–12-month-old children has probably only been achieved with the widespread use of fortified packaged complementary foods (FPCF)^(^ ^10^ ^)^.

Fortification of staple foods cannot provide sufficient nutrients to small children as staple foods are fortified at low levels for the safety of the overall population. The limited quantities of these foods consumed by 6–23-month-old children are thus insufficient to meet their micronutrient needs^(^ ^8^ ^)^. Supplementation is well suited for targeting specific populations. However, compliance is often low^(^ ^11^ ^)^.

FPCF, on the other hand, are characterized by standardized micronutrient content tailored to the needs of young children. Moreover, packaged foods can use cheap and well-developed distribution networks. This is especially important for complementary foods for young children as they need to be bought on a regular basis. Easy availability was found to improve uptake^(^ ^12^ ^)^. Two recent reports by the Global Alliance for Improved Nutrition (GAIN) further emphasize the importance of convenience, speed of preparation and taste for the uptake of complementary infant foods^(^ ^13^ ^,^ ^14^ ^)^.

Cost-effectiveness evaluations inform decision makers on whether the funds available are well spent. An intervention is considered cost-effective when the monetary cost per unit of outcome is below a predetermined threshold value.

We estimated the cost-effectiveness of subsidies on FPCF for the reduction of iodine deficiency (IoD)^(^ ^15^ ^)^, iron-deficiency anaemia (IDA) and vitamin A deficiency (VAD) in 6–23-month-old children in Pakistan. For this purpose, we built a health economic model of the social cost of IoD, IDA and VAD, and carried out a survey among 1764 Pakistani households to assess their price sensitivity of demand.

## Methods

### Overview of study design

**Fig. 1:**
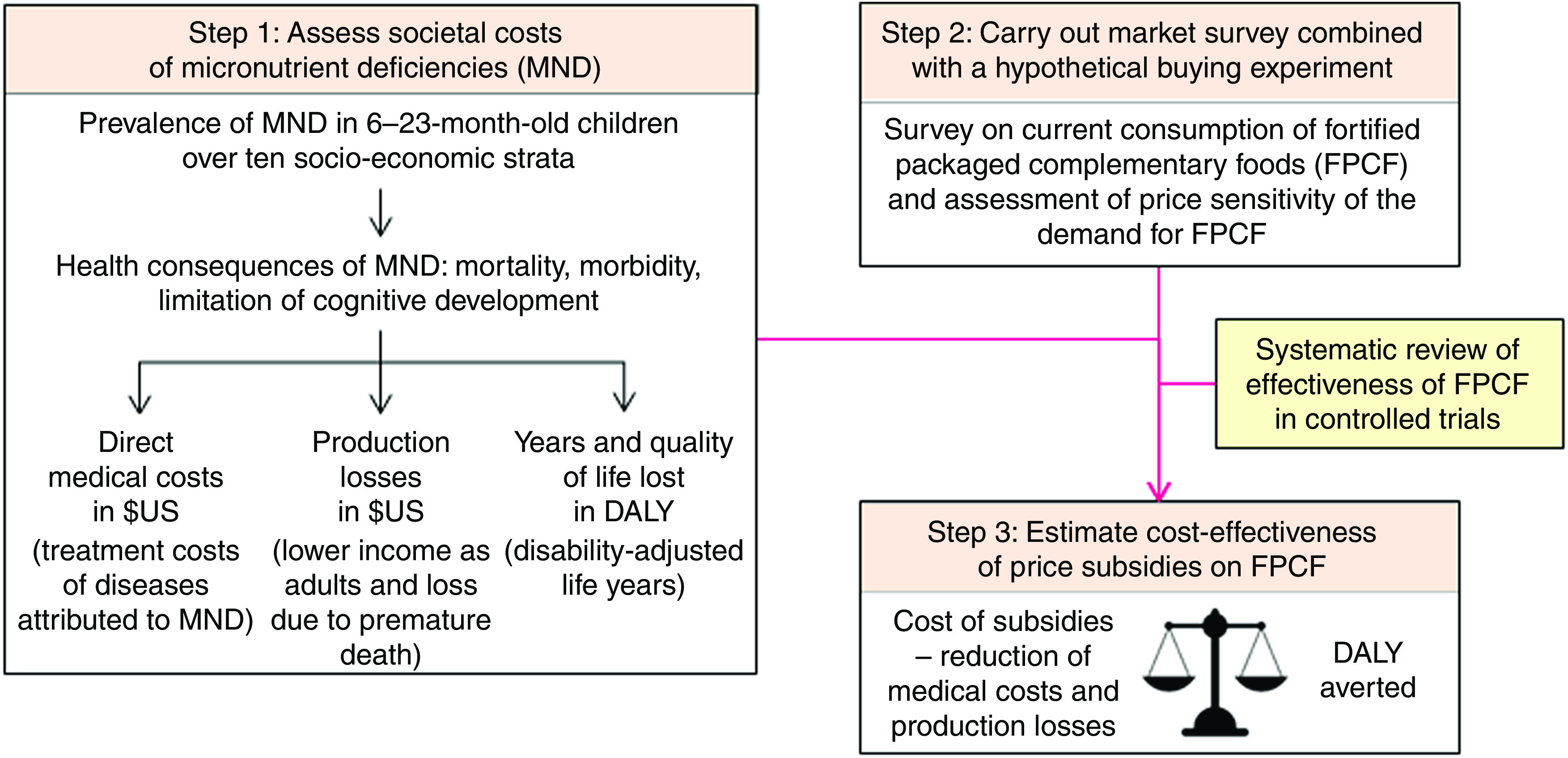
Overview of the model linking micronutrient deficiencies to health economic outcomes

The present study estimated the effectiveness and cost-effectiveness of subsidies on FPCF for the reduction of MND in 6–23-month-old Pakistani children following the approach developed in Plessow *et al*.^(^ ^16^ ^)^. Figure 1 illustrates how the study combined three main steps. First, it adapted a previous model of the societal costs of MND in Pakistan^(^ ^17^ ^)^ to the districts of Faisalabad and Hyderabad. Second, a survey was conducted to assess the current consumption of FPCF in these districts and the reaction to hypothetical price discounts. Third, the results of the first and second steps were combined with a systematic review on the effectiveness of FPCF in reducing MND^(^ ^18^ ^)^. Cost-effectiveness was measured by the net societal cost per disability-adjusted life year (DALY) averted. Net social costs were determined by subtracting the averted medical costs and production losses from the price subsidies.

### Societal cost of micronutrient deficiencies

We calculated the current lifetime costs of IoD, IDA and VAD in a birth cohort of children affected by these deficiencies between the ages of 6 and 23 months. These costs were calculated by adapting the model of the societal costs of MND in Pakistan^(^ ^17^ ^)^ to the districts of Faisalabad and Hyderabad^(^ ^19^ ^)^. The health and costs consequences of MND in this age group are described in detail in the online supplementary material (Section 1, Tables S1 and S2). In summary, IoD may lead to mild to severe cognitive impairment measured in loss of IQ (intelligence quotient) points^(^ ^15^ ^,^ ^20^ ^)^. IDA may lead to impaired cognitive development and physical activity, while severe IDA may additionally lead to increased all-cause mortality^(^ ^21^ ^–^ ^23^ ^)^. VAD may lead to Bitot’s spots, night blindness, diarrhoea, measles and all-cause mortality^(^ ^24^ ^)^. Permanent cognitive impairment and premature mortality lead to wage losses and DALY, whereas reversible cognitive impairment leads to current DALY only^(^ ^17^ ^)^. Impaired physical activity, Bitot’s spots, night blindness, diarrhoea and measles can be reversed with micronutrient supplementation (as soon as the deficiency disappears) and thus lead to current DALY only.

We assumed that children in the two districts were distributed across socio-economic status (SES) strata according to the province-level data of the Demographic and Health Survey (DHS) 2012–13^(^ ^19^ ^)^. The same approach was applied to SES- and age-specific mortality rates, as well as to the incidence of measles and diarrhoea. The prevalence rates of IoD, IDA and VAD were calculated based on the National Nutrition Survey (NNS) 2011^(^ ^1^ ^)^. The prevalence of IoD was calculated for school-age children (SAC), using the established WHO cut-offs^(^ ^15^ ^)^, as the NNS does not provide spot urinary iodine concentration (UIC) values for 6–23-month-old children^(^ ^25^ ^)^. The calculation of the population-attributable fraction of mental impairment due to IoD was based on Zimmermann and Andersson^(^ ^20^ ^)^ and is described in detail in the appendix of Wieser *et al*.^(^ ^17^ ^)^. The treatment costs of diarrhoea and measles were assessed in a household survey.

### Household survey and hypothetical buying experiment

To assess the current consumption of FPCF and the price sensitivity of demand for FPCF, we carried out a household survey. The survey took place between July and September 2015 in the districts of Faisalabad and Hyderabad located in the provinces of Punjab and Sindh. These are the two most populous provinces of Pakistan with a share of 46 % and 28 %, respectively, of the total population of 200 million.

Households were selected using a stratified two-stage sample design based on the Pakistan Bureau of Statistics’ sampling frame. In a first step we randomly selected forty-five primary sampling units, each comprising 200–250 households. In a second step we randomly selected twenty-five households with at least one 6–23-month-old child within each of the selected primary sampling units, with stratification by three wealth classes (low, middle, high). All interviews were conducted with the decision maker responsible for food and beverage purchases.

The survey questionnaire (see online supplementary material, Section 2) was composed of six parts assessing: (i) household composition; (ii) current buying and serving behaviour of FPCF; (iii) price sensitivity of demand for FPCF; (iv) nutritional knowledge and attitudes regarding child nutrition; (v) occurrence and treatment of specific diseases like diarrhoea and acute respiratory infection; and (vi) socio-economic characteristics of the household. These socio-economic characteristics assessed in the survey were used to calculate a common wealth index between our survey, the NNS^(^ ^1^ ^)^ and the DHS^(^ ^26^ ^)^. Based on this wealth index, households were classified into ten socio-economic strata (SES wealth index deciles) allowing us to link the information collected in the survey with data from the NNS^(^ ^1^ ^)^ and the DHS^(^ ^19^ ^)^.

The price sensitivity of demand for FPCF was assessed with a hypothetical marketing experiment:

1. Respondents were asked to imagine that they were eligible for a programme allowing them to buy FPCF at a constantly discounted price until the child turns 2 years of age.
2. Respondents were then asked whether they would buy these FPCF at a discount of 20 %, 40 %, 60 % or 80 %, which had been randomly assigned in advance. If the respondent was currently buying FPCF, the discount related to the brand and market price of the FPCF currently bought. If the respondent was currently not buying FPCF, the discount related to de-branded 25 g sachets of FPCF (see online supplementary material, Fig. S1), which were shown together with information on the recommended amount and duration of feeding.
3. If the respondents were willing to buy the FPCF at the discounted price, they were asked to state the amount of FPCF they would buy in a regular week. If respondents were not willing to buy at the discounted price, the next higher discount was offered repeatedly until the highest discount of 80 % was reached.

To avoid anchoring effects, we used only the information on the first drawn discount to estimate the price sensitivity of demand. We also applied an *ex post* correction (see online supplementary material, Section 3) which has been shown to be effective in reducing hypothetical bias^(^ ^27^ ^)^.

Based on the marketing experiment we classified households as ‘current buyers’, ‘potential buyers’ and ‘non-buyers’. Current buyers included the households which had bought FPCF in the 7d prior to the interview. Potential buyers included the households which had not bought FPCF in the days before the interview but claimed they would do so at a discount of 20 % to 80 %. Non-buyers included the households which had not bought FPCF in the days before the interview and claimed that they would not buy them even at a discount of 80 %.

### Cost-effectiveness estimation of price subsidies

The cost-effectiveness of subsidies on FPCF was estimated in three steps. First, we estimated the demand effects of price subsidies. Second, we translated these demand effects into health effects using the results of a systematic review of randomized controlled trials on the effectiveness of FPCF in reducing IDA and VAD and applying the RDA of iodine for IoD. Third, we calculated the cost-effectiveness of the subsidies by running our model of the lifetime costs of IoD, IDA and VAD in 6–23-month-old children with and without the subsidies.

The demand effects of price subsidies were estimated with a first difference model. This approach solves the typical identification problems associated with a cross-section estimation of demand. These problems were likely to be severe, because most of the variation in price was due to product variation. If wealthier households bought both larger quantities and higher-quality products, a cross-section estimation could lead to an upwards sloping demand curve, because the price effect cannot be separated from the effects of unobserved preferences. Furthermore, the first difference model mitigates potential problems arising from the failed randomization as it eliminates all unobserved time-invariant individual effects that are possibly correlated with the price of FPCF.

We estimated slightly different models depending on the type of household. A log-linear model was applied for current buyer households (equation (1)) and a linear model for potential buyer households (equation (2)).* We thus estimated the following models:1

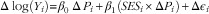

and2

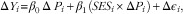

where Δ*Y*_*i*_=*Y*_*i*1_−*Y*_*i*2_, with *Y*_*i*1_ representing the weekly amount of FPCF bought by household *i* at current market prices (in grams), *Y*_*i*2_ representing the hypothetical demand at discounted prices and Δ*Y*_*i*_ representing the hypothetical change in demand as a result of the price discount. Similarly, 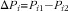 , with *P*_*t*1_ being the price for 25 g of FPCF (the size of one serving) paid by household *i*, *P*_*i*2_ being the randomly drawn lower price and Δ*P*_*i*_ being the size of the price discount. As we expected the response in demand to price changes to depend on wealth, we included an interaction term between price and wealth, represented by *SES*_*i*_, an ordinal variable indicating the household’s wealth index decile. The parameters of interest therefore are *β*_0_ and *β*_1_, capturing the partial effect of price changes overall and depending on wealth, respectively, on changes in demand. *ϵ_i_* represents an error term at household level. Note that *SES*_*i*_ drops out by taking first differences.

Different robustness tests were performed. We first compared the estimates of the parametric model as described in equations (1) and (2) with the estimates of a semi-parametric model including ten dummy variables for SES and found that the variation in the demand effect across SES is modelled fairly appropriately with our baseline model assuming constant effects. We also considered different limited dependent variable models and evaluated model performance based on 1000 cross-validated out-of-sample predictions. The parametric fixed-effects model performed best (see online supplementary material, Section 4, Fig. S2 and Table S3). Models including higher polynomials in price as well as the log price were also tested. However, as we did not find any significant improvements in the overall goodness-of-fit of the model (as measured by adjusted *R* ^2^, Akaike information criterion and Bayesian information criterion), they were rejected.

To translate demand effects into health effects we drew on a systematic review of randomized controlled trials on the effectiveness of FPCF in reducing IDA and VAD in young children^(^ ^18^ ^)^. The review shows that the daily provision of FPCF compared with non-fortified packaged complementary foods leads to an increase in Hb level of 8·7 (95 % CI 5·7, 11·6) g/dl and an increase in serum retinol level of 3·7 (95 % CI 1·3, 6·1) μg/dl. We assumed that daily provision of 75 g of FPCF would have an identical effect on Hb and serum retinol levels as the average effects assessed in the systematic review. This amount of 75 g is higher than the average amount of cereals provided in the studies included in the systematic review. However, three 25 g packages of the fortified infant cereals currently available in Pakistan contain approximately the same amount of iron provided on average in the systematic review. For the effectiveness of FPCF in reducing IoD, we assumed that the recommended daily dose of 75 g of FPCF would contain the WHO RDA of iodine of 90 μg/d^(^ ^15^ ^)^. We calculated the respective change in UIC based on the following equation proposed by the US Institute of Medicine^(^ ^28^ ^)^ and applied the UIC cut-offs for mild, moderate and severe IoD as defined by WHO for SAC^(^ ^15^ ^)^:3

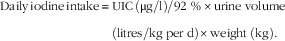




**Fig. 2:**
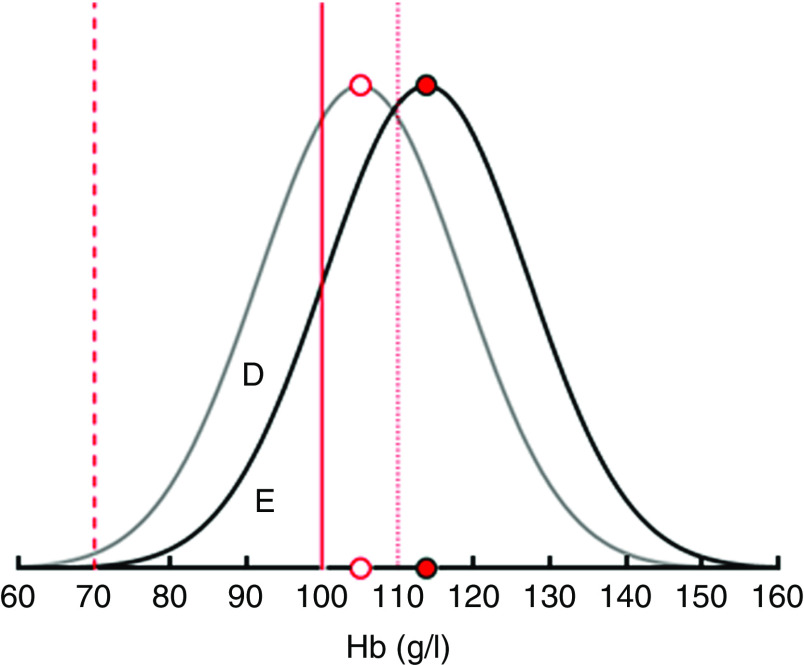
Translating the demand effects into changes in iron-deficiency anaemia (IDA) prevalence among 6–23-month-old children from Faisalabad and Hyderabad districts, Pakistan. The figure shows that an increase in the consumption of fortified packaged complementary foods by 75 g (the recommended daily dose) shifts the Hb distribution to the right by 8·7 g/l and thus reduces the share of children with IDA by the area D. Area E represents the share of children with IDA who remain deficient even after the maximum intervention (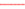 , limit mild anaemia; 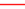 , limit moderate anaemia; 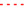 , limit severe anaemia; 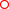 , average Hb before intervention; 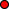 , average Hb with intervention)

Figure 2 uses IDA as an example to illustrate how the demand effects are translated into changes in IoD, IDA and VAD prevalence in a single SES. An intervention increasing the consumption of FPCF by 75 g would shift the Hb distribution to the right by 8·7 g/dl. As a result, the prevalence of IDA would decrease. Changes in consumption that are less than the recommended daily dose are assumed to increase Hb levels proportionately.

The cost-effectiveness of an intervention was calculated by first computing the net social costs of an intervention. These net social costs correspond to the cost of the subsidies minus the net present value of future production losses in terms of lower future adult wage. The net social costs of the intervention were then divided by the amount of DALY averted to obtain the cost-effectiveness ratio (see online supplementary material, Section 5 and Fig. S3, for illustrative examples). DALY averted are the difference between the pre- and post-intervention DALY. DALY correspond to the sum of life years lived with disability and life years lost due to premature mortality. They depend on the prevalence of MND, which is evaluated with the biomarker of each micronutrient and its respective WHO threshold value.

### Sensitivity analyses

To test the robustness of the results, we ran a probabilistic and univariate sensitivity analysis. The probabilistic sensitivity analysis creates a range of possible model outcomes and determines the CI of the main model outcomes. The univariate sensitivity analysis shows the impact of a possible overestimation of the IoD prevalence. The results of these sensitivity analyses are reported in the online supplementary material (Section 8, Tables S5 and S6, Figs S6 to S8).

## Results

### Societal costs of micronutrient deficiencies

We first calculated the lifetime costs of a birth cohort of children in the districts of Hyderabad and Faisalabad affected by IoD, IDA and VAD between the ages of 6 and 23 months. The birth cohort included nearly half a million children. The number of children per SES decreased with increasing wealth (Table 1). Mortality rates decreased by a factor of ten from the lowest to the highest SES.

**Table 1:** Distribution of birth cohort and mortality rate by socio-economic status (SES wealth index decile) among 6–23-month-old children from Faisalabad and Hyderabad districts, Pakistan

	SES1 (low)	SES2	SES3	SES4	SES5	SES6	SES7	SES8	SES9	SES10 (high)	Cohort size (in 1000s)
Share of birth cohort (%)	12·1	10·5	8·8	10·4	8·7	9·8	11·3	10·1	9·6	8·7	487·4
Yearly mortality rate (per 1000 children)	19·9	17·9	15·9	13·9	11·9	9·9	8·0	6·0	4·0	2·0	

Authors’ calculations based on Demographic and Health Survey 2012–13 data, 2010 and 2011 population data^(^ ^19^ ^,^ ^44^ ^,^ ^45^ ^)^.

**Fig. 3:**
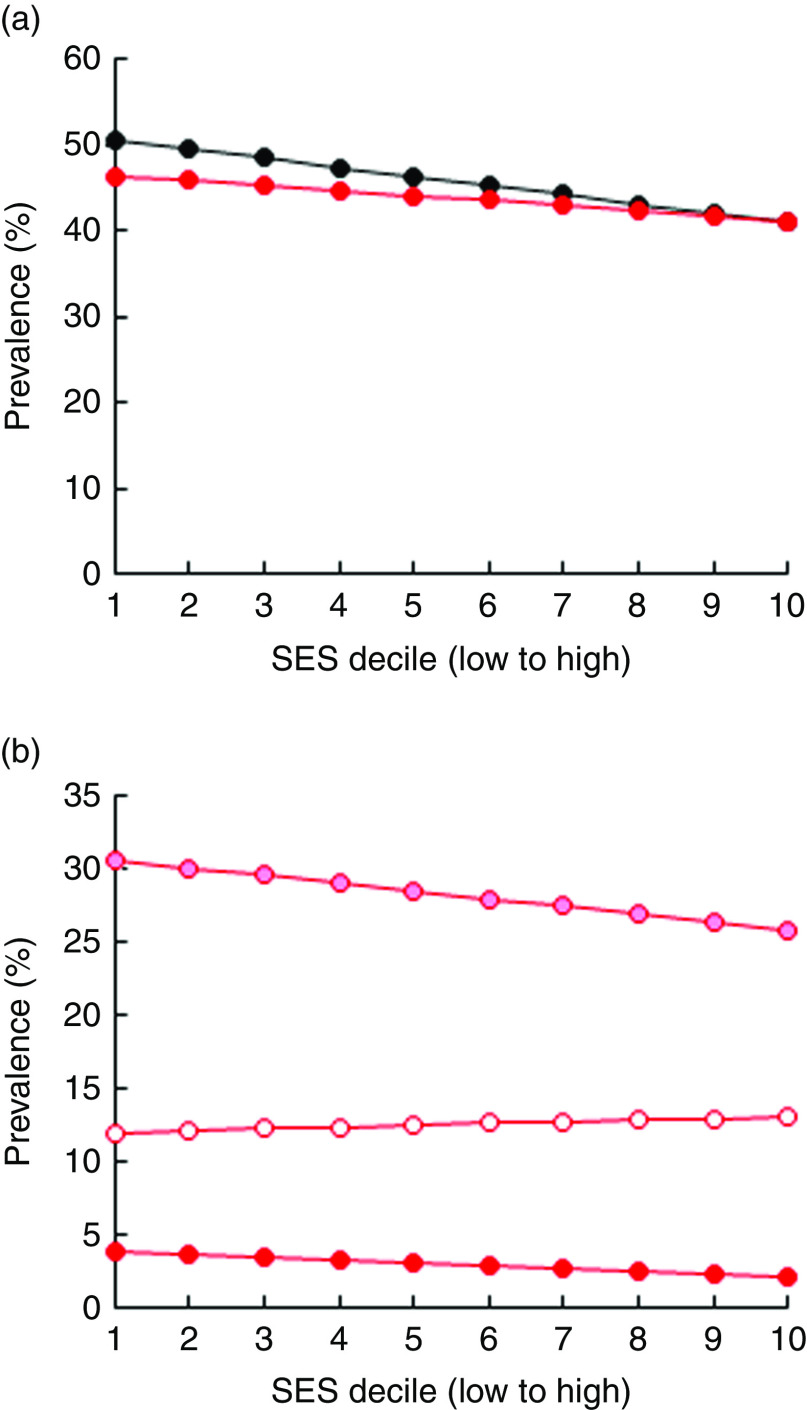
Prevalence of (a) iron-deficiency anaemia (IDA; 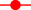 ) and vitamin A deficiency (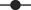 ) and (b) moderate IDA (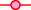 ), mild IDA (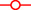 ) and severe IDA (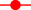 ) by socio-economic status (SES wealth index decile) among 6–23-month-old children from Faisalabad and Hyderabad districts, Pakistan (authors’ calculation based on Hb and serum retinol level data from the National Nutrition Survey 2011^(^ ^1^ ^)^)

Figure 3 illustrates the prevalence of VAD and IDA across SES. The prevalence of VAD decreased from 51 % in the poorest to 41 % among the wealthiest SES, while the corresponding prevalence of IDA decreased from 46 % to 41 % (Fig. 3(a)). Moderate and severe IDA decreased with wealth whereas mild IDA increased slightly (Fig. 3(b)). Due to data limitations, the baseline prevalence rate of IoD was not stratified by SES. The overall prevalence of IoD among SAC in Pakistan was almost 40 %, while the prevalence of mild, moderate and severe IoD was at 26 %, 11 % and 2 %, respectively^(^ ^1^ ^)^.

**Fig. 4:**
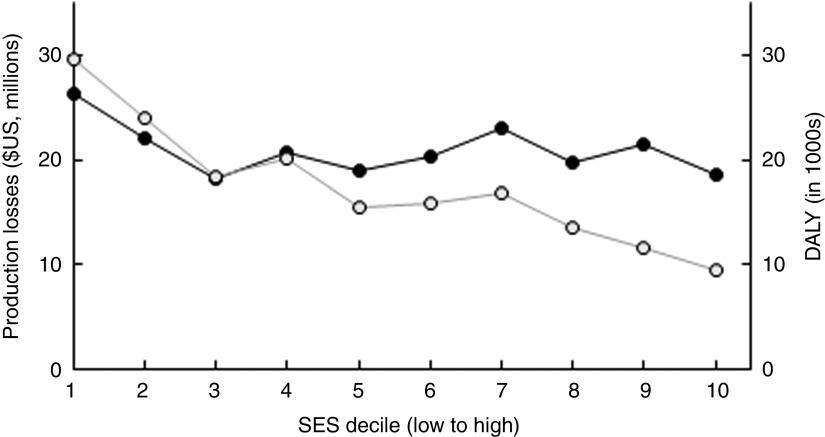
Distribution of costs (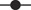 , production losses; 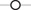 , disability-adjusted life years (DALY)) of the three micronutrient deficiencies by socio-economic status (SES wealth index decile) among 6–23-month-old children from Faisalabad and Hyderabad districts, Pakistan (authors’ calculation)

Total societal costs amounted to monetary costs of $US 209 million and 175 000 DALY. Table 2 reports costs by type as well as by time period in which they arise. Costs were mainly driven by future production losses resulting from impaired cognitive development due to IDA and IoD. While DALY decreased considerably with increasing wealth, production losses decreased only slightly (Fig. 4).

**Table 2:** Societal costs of iron-deficiency anaemia (IDA), vitamin A deficiency (VAD) and iodine deficiency (IoD) in 6–23-month-old children from Faisalabad and Hyderabad districts, Pakistan

				DALY (in 1000s)			
	Production losses ($US, millions)			Current	Future	Mortality	Total
	Future	Mortality	Total	YLD	YLD	YLL	DALY
IDA	153	6	159	10	93	14	117
VAD	–	21	21	1	–	47	48
IoD	28	–	28	1	10	–	10
Total	182	27	209	11	103	61	175

YLD, years lived with disability; YLL, years of life lost. The production losses and DALY do not overlap, hence reporting both does not lead to double counting^(^ ^46^ ^,^ ^47^ ^)^. Future production losses occur during the future working life. Current DALY occur in the 6–23-month period.

### Results of the market survey

In total, 1764 households with a child aged 6–23 months were contacted. Only eighty (4·5 %) of these households refused to participate, leading to an initial sample of 1684 interviews. Quality and plausibility checks led to the exclusion of thirty-one (1·8 %) observations, seventeen because of implausible values on quantity bought or prices paid and fourteen because of missing information on hypothetical demand at discounted prices. Another 112 (6·7 %) observations were excluded due to the *ex post* correction applied to the hypothetical questions in the marketing experiment. Their exclusion should not induce any selection bias as our checks showed no significant differences between included and excluded observations in any of the characteristics except for nutritional knowledge.* Table 3 shows the number of interviews and the final sample size by wealth strata and district.

**Table 3:** Sample size and area coverage

District	Interviews (*n*)	Final sample (*n*)
Faisalabad		
Low wealth	268	249
Middle wealth	271	242
High health	275	262
Total	814	753
Hyderabad		
Low wealth	284	256
Middle wealth	300	265
High health	286	267
Total	870	788
Overall	1684	1541

The table shows unweighted numbers of observations. Authors’ survey of households with 6–23-month-old children from Faisalabad and Hyderabad districts, Pakistan.

Table 4 gives an overview over the characteristics of the surveyed households by wealth quintile. Table 4(a) shows that nearly 80 % of the households in the wealthiest quintile were located in Faisalabad, while the lower quintiles were more balanced with respect to district. Mothers were the main decision makers regarding food choices for the child and their share increased slightly with wealth. Virtually all mothers were married and between 86 % and 94 % worked at home. The share of illiterate mothers decreased from 49 % in the lowest to 3 % in the highest quintile, while the share with a tertiary education increased from 1 % to 57 %. Wage inequality was remarkable, with monthly household earnings being eighteen times higher in the wealthiest compared with the poorest 20 % of the households.

**Table 4:** Characteristics and current consumption behaviour, by socio-economic status (SES wealth index decile), in households with 6–23-month-old children from Faisalabad and Hyderabad districts, Pakistan

	SES decile									
	SES1–2		SES3–4		SES5–6		SES7–8		SES9–10	
	Mean	sd	Mean	sd	Mean	sd	Mean	sd	Mean	sd
(a) Socio-economic characteristics										
Hyderabad district	0·47	0·5	0·52	0·5	0·52	0·5	0·42	0·5	0·18	0·5
Mother is decision maker in food for child	0·85	0·4	0·82	0·4	0·90	0·3	0·93	0·3	0·92	0·3
Mother is married	1·00	0·1	1·00	0·1	1·00	0·0	1·00	0·0	1·00	0·0
Education of mother										
Illiterate	0·49	0·5	0·24	0·4	0·12	0·3	0·04	0·2	0·03	0·2
Up to 9 years of school	0·34	0·5	0·27	0·5	0·21	0·4	0·17	0·4	0·04	0·2
10th class	0·11	0·3	0·29	0·5	0·32	0·5	0·25	0·4	0·13	0·3
11th, 12th class	0·06	0·2	0·12	0·3	0·19	0·4	0·21	0·4	0·23	0·4
(Post) graduate	0·01	0·1	0·08	0·3	0·16	0·4	0·33	0·5	0·57	0·5
Mother is not working/homeworker	0·86	0·4	0·94	0·2	0·95	0·2	0·96	0·2	0·94	0·2
Monthly household earnings ($US)	169	119	272	450	418	297	1373	2142	3032	2522
Age of the child (months)	13·7	4·8	13·5	4·9	13·6	4·9	13·9	5·1	14·2	4·9
Gender of the child: boy	0·53	0·5	0·53	0·5	0·53	0·5	0·45	0·5	0·51	0·5
(b) Nutritional knowledge										
Received advice regarding child feeding	0·52	0·5	0·59	0·5	0·68	0·5	0·73	0·5	0·88	0·3
Heard about iron-deficiency anaemia?	0·29	0·5	0·45	0·5	0·51	0·5	0·72	0·5	0·86	0·4
Heard about vitamin A deficiency?	0·18	0·4	0·31	0·5	0·37	0·5	0·54	0·5	0·79	0·4
Heard about iodine deficiency?	0·32	0·5	0·51	0·5	0·63	0·5	0·73	0·4	0·91	0·3
Using iodized salt?	0·22	0·4	0·34	0·5	0·48	0·5	0·68	0·5	0·81	0·4
(c) FPCF current consumption										
Current buyers (proportion)	0·22	0·4	0·37	0·5	0·47	0·5	0·44	0·5	0·53	0·5
Quantity bought (g/week)	190	191	171	152	186	160	210	248	174	145
Price paid per 25 g (PKR)*	18·8	5·2	19·2	5·1	19·4	4·8	21·5	5·0	22·8	4·3
Current non-buyers (proportion)	0·78	0·4	0·62	0·5	0·53	0·5	0·56	0·5	0·47	0·5
(d) Selected reasons for not buying (as proportion of current non-buyers, multiple answers possible)										
Not affordable	0·41	0·5	0·25	0·4	0·12	0·3	0·05	0·2	0·00	0·0
Not healthy	0·07	0·3	0·07	0·3	0·06	0·2	0·10	0·3	0·17	0·4
Child doesn’t like it/digestive problems	0·35	0·5	0·38	0·5	0·42	0·5	0·48	0·5	0·55	0·5
Homemade food is better (as to variety)	0·10	0·3	0·13	0·3	0·20	0·4	0·33	0·3	0·55	0·5
Relatives, friends, doctors are against it	0·05	0·2	0·04	0·2	0·07	0·3	0·05	0·2	0·04	0·2
Not available	0·00	0·0	0·01	0·1	0·01	0·1	0·01	0·1	0·00	0·0
(e) Hypothetical experiment (as proportion of current non-buyers)										
Would serve FPCF after having received product information and if it were for free†	0·92	0·2	0·89	0·3	0·94	0·2	0·98	0·1	1·00	0·0
Buying at 20–80 % discount (potential buyers)	0·53	0·5	0·41	0·5	0·37	0·5	0·32	0·5	0·06	0·2
Not buying at 20–80 % discount (non-buyers)	0·47	0·5	0·59	0·5	0·63	0·5	0·68	0·5	0·94	0·2
Number of observations	339		322		323		324		345	

FPCF, fortified packaged complementary foods. The table shows weighted means and sd of selected characteristics for households separated by wealth quintiles from lowest (SES1–2) to highest (SES9–10). If not indicated otherwise, the mean refers to the whole sample size (parts (a) and (b)). Observations were weighted according to district size.

* $US 1=101·565 PKR (Pakistani Rupee).

† Accompanying information: ‘Many children suffer from micronutrient deficiencies and therefore cannot develop optimally. Suppose you were eligible for a programme allowing you, as the mother of a child aged 6–23 months old, to buy packaged infant cereal at a reduced price. These infant cereals are a nutritious baby food containing added minerals and vitamins. They support the healthy growth and development of your child. The added minerals and vitamins may help protect your child from infections, eye problems, weak memory and ill health in general.’

Table 4(b) shows that wealthier households were more often aware of MND, that they were more likely to have received professional advice in terms of healthy child nutrition and that they were more likely to use iodized salt.

Table 4(c) illustrates the current consumption of FPCF. The share of current buyers increased markedly with wealth. While only one in five of the poorest 20 % of households was serving FPCF, about one in two did so in the wealthiest 20 %. The relationship between wealth and quantities served did not seem to follow a specific pattern. The weekly quantities varied from 171 g in SES3–4 to 210 g in SES7–8, corresponding to a share of 32–40 % of the recommended daily dose. Prices paid for FPCF increased slightly with wealth, as wealthier households bought more expensive products. The share of current non-buyers (including potential buyers and non-buyers as defined in the ‘Methods’ section) decreased from 78 % in SES1–2 to 47 % in SES9–10.

Table 4(d) reports the reasons for not buying FPCF stated by current non-buyers, giving an indication of what might motivate them to buy FPCF. Financial constraints were the main reason for not buying FPCF among poorer households (41 % in SES1–2 and 0 % in SES9–10). Wealthier households, although better informed about MND (compare with Table 4(b)), mainly stated that homemade food was better or even that FPCF were not healthy (7 % in SES1–2 and 17 % in SES9–10). Digestive problems and taste as reasons for not buying were mentioned more often by wealthier than by poorer households (55 % in SES9–10 *v*. 35 % in SES1–2).

**Fig. 5:**
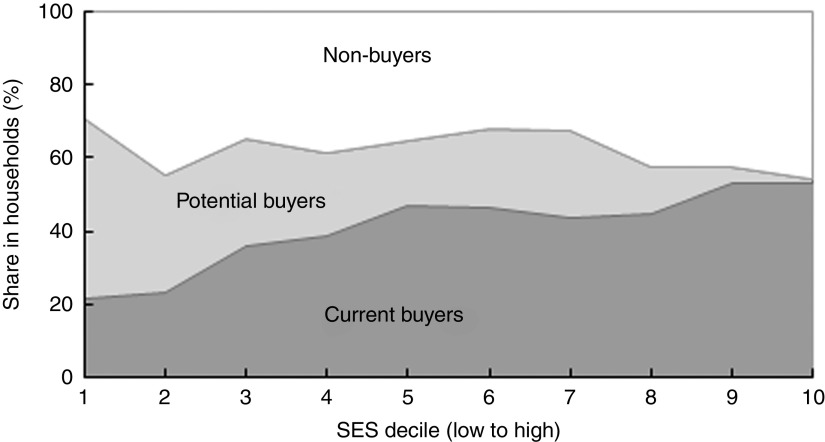
Types of buyer of fortified packaged complementary foods by socio-economic status (SES wealth index decile) in households with 6–23-month-old children from Faisalabad and Hyderabad districts, Pakistan (authors’ survey)

However, providing current non-buyers with a short description of the product and its health benefits prior to the marketing experiment increased the share of people willing to serve FPCF if they were for free to 94 % on average and even more among wealthier households (Table 4(e)). This is a clear indication that price-based interventions along with product information for current non-buyers might be an effective way to increase the demand for FPCF. Table 4(e) also shows that the proportion of households buying at a 20–80 % discount (potential buyers) was considerably lower than if FPCF were provided for free. The proportion decreased from 53 % in SES1–2 to 6 % in SES9–10. Figure 5 illustrates the distribution of buyer types across the wealth deciles (see online supplementary material, Section 6 and Table S4 for detailed results of the buying experiment).

### Estimation of price sensitivity

Table 5 shows the point estimates for the models specified in equations (1) and (2). The sign on the overall price effect of demand is negative for both current and potential buyers, indicating that quantity demand increased as price decreased. However, the sign of the interaction effect differs between the two groups. In contrast to the non-buyer households, the demand effects decreased with wealth among the households that already bought FPCF.

**Table 5:** Point estimates and marginal demand effects in households with 6–23-month-old children from Faisalabad and Hyderabad districts, Pakistan

	Log(demand)		Demand (g/week)	
	Current buyers		Potential buyers	
Dependent variable	Mean	se	Mean	se
(a) Point estimates				
Price (per 25 g)	−0·058*	0·009	−9·33*	2·18
Price (per 25 g)×SES	0·003*	0·001	−1·71*	0·53
Mean current demand	185·9		0·00	
Mean price paid (per 25 g)	20·6		0·00	
Number of observations	610		297	
Adjusted *R* ^2^	0·153		0·161	
*P* value (*F* statistic)	0·00		0·00	
(b) Marginal effects (g/week)				
mfx_SES1_	10·47*	1·59	11·03*	1·77
mfx_SES2_	9·92*	1·39	12·74*	1·46
mfx_SES3_	8·61*	1·09	14·45*	1·30
mfx_SES4_	7·52*	0·86	16·15*	1·35
mfx_SES5_	7·50*	0·76	17·86*	1·59
mfx_SES6_	7·56*	0·69	19·56*	1·95
mfx_SES7_	8·65*	0·78	21·27*	2·37
mfx_SES8_	5·92*	0·60	22·98*	2·84
mfx_SES9_	5·60*	0·72	24·68*	3·32
mfx_SES10_	4·02*	0·70	26·39*	3·82

SES, socio-economic status; FPCF, fortified packaged complementary foods; PKR, Pakistani Rupee. The table shows the results of the first difference model as specified in equations (1) and (2). Part (a) reports the point estimates of the price effect and the price effect by SES on the quantity demanded. Part (b) shows the marginal demand effects for a price decrease of a 25 g sachet of FPCF by 1 PKR (corresponding to an increase of 4–5 %).

* Denotes statistical significance on the 1 % level.

The marginal demand effects with respect to price by SES denote the change in the weekly demand for FPCF (in grams) in response to a price decrease of 1 PKR (Pakistani Rupee; $US 1=101·565 PKR) per 25 g (the size of one serving). The weekly demand of the poorest decile of households would, for example, increase by 10·47 g with a price decrease of 1 PKR. With increasing wealth, the marginal demand effects diverged between current buyers and potential buyers. Price discounts were most effective for low SES among current buyers, while they were most effective for high SES among potential buyers. The share of high-SES potential buyers was however very small. The price elasticity of demand could be calculated only for current buyers and ranged from −0·96 for SES1 to −0·60 for SES10.

**Fig. 6:**
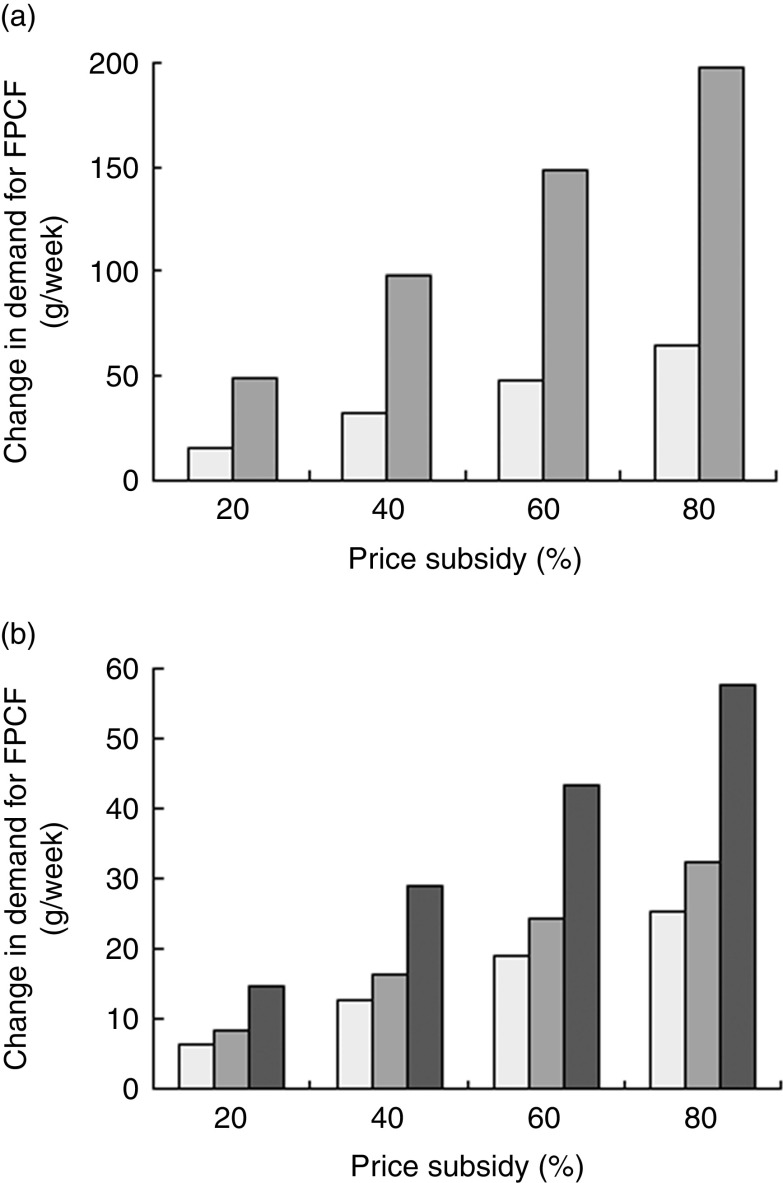
(a) Estimated demand effects and (b) weighted average demand effects of price subsidies on weekly demand for fortified packaged complementary foods (FPCF) across buyer types (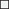 , current buyers; 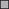 , potential buyers; 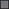 , all households) in households with 6–23-month-old children from Faisalabad and Hyderabad districts, Pakistan (authors’ calculation)

Figure 6 presents the estimated demand effects for different price subsidies. The average potential buyer responded more strongly in absolute terms than the average current buyer household (Fig. 6(a)). A price subsidy of 20 % would, for instance, lead to an increase in the weekly quantity bought by 50 g for potential buyers and by 16 g for current buyers. Figure 6(b) shows the weighted average demand effects for the population as a whole, as well as separately for current and potential buyer households. An 80 % subsidy led to an overall average increase of 58 g/week per household (including non-buying households). Although the differences between current and potential buyers were smaller when considering population shares, potential buyers still responded stronger to price subsidies.

### Effectiveness of subsidies

We calculated the effectiveness and cost-effectiveness for different interventions differing in the size of the subsidy (20 % and 80 %) as well as in the share of households eligible for the subsidy. Interventions spanned from a 20 % price subsidy for the poorest 10 % of the households to an 80 % price subsidy for the whole population. We also considered interventions that provided all children within a wealth decile with the recommended dose of 75 g FPCF/d.

Table 6 shows the effectiveness of these interventions with respect to the percentage of DALY averted. A 40 % subsidy for the poorest 20 % (SES2) would for instance avert 1·8 % of DALY. Increasing the highest SES eligible for a subsidy, increased the share of DALY averted. However, the intervention becomes relatively less effective when extending the intervention to higher SES as the share of non-buyer households increases with wealth and the prevalence of MND is lower in the higher SES. Increasing the subsidy, on the other hand, leads to an approximately proportionate increase in DALY averted.

**Table 6:** Disability-adjusted life years (DALY) caused by iron-deficiency anaemia, vitamin A deficiency and iodine deficiency that are averted by the price subsidy on fortified packaged complementary foods among 6–23-month-old children from Faisalabad and Hyderabad districts, Pakistan

	Highest SES decile eligible									
	SES1	SES2	SES3	SES4	SES5	SES6	SES7	SES8	SES9	SES10
(a)										
20 % subsidy	0·5	0·9	1·3	1·7	2·0	2·3	2·8	3·1	3·3	3·4
30 % subsidy	0·8	1·3	1·8	2·3	2·8	3·3	4·0	4·5	4·8	4·9
40 % subsidy	1·1	1·8	2·5	3·1	3·7	4·4	5·4	5·9	6·3	6·5
50 % subsidy	1·3	2·2	3·1	4·0	4·7	5·6	6·7	7·4	7·9	8·1
60 % subsidy	1·5	2·6	3·6	4·6	5·4	6·5	7·8	8·6	9·2	9·5
70 % subsidy	1·7	3·0	4·1	5·3	6·2	7·4	8·9	9·8	10·5	10·8
80 % subsidy	1·9	3·3	4·6	5·9	6·9	8·2	9·9	10·9	11·6	12·0
(b)										
75 g/d for free	5·0	8·4	11·5	14·8	17·5	20·4	23·6	26·0	28·2	29·9
75 g/d for free if no non-buyers	6·5	11·8	16·0	20·7	24·4	28·3	32·5	36·0	39·1	41·8
% non-buyers	29·3	44·8	34·6	38·8	35·3	31·9	32·6	42·4	42·8	45·9

SES, socio-economic status; FPCF, fortified packaged complementary foods. Part (a) shows the percentage of DALY averted by interventions that differ in the size of the price subsidy and the SES eligible. Note that the values are cumulative as the columns represent the highest SES eligible. Part (b) shows the results for two ‘for free’ scenarios. The first row shows the results for a scenario in which all 6–23-month-old children living in a certain SES would be given 75 g FPCF/d for free in addition to the amount they already consumed. The last row of part (b) goes one step further by assuming 100 % compliance, i.e. that there are no non-buyer households. These results thus represent the maximum possible effect.

We also evaluated two ‘for free’ scenarios to assess the maximum possible effect. The first row in Table 6(b) shows the effect of an intervention providing all 6–23-month-old children in current and potential buyer households with 75 g FPCF/d for free, under the assumption that these 75 g/d were consumed in addition to the amount already consumed. The second row shows the maximum possible effect that could be achieved if all children, including those in non-buyer households, received 75 g FPCF/d for free. This intervention would avert a maximum of 41·8 % of DALY. These scenarios mainly serve as comparison to the discounts because, although they are highly effective, they also come along with very high intervention costs (as shown in Table 7).

**Table 7:** Cost-effectiveness of different interventions with fortified packaged complementary foods (FPCF) for one birth cohort of 6–23-month-old children from Faisalabad and Hyderabad districts, Pakistan

Intervention	SES eligible	Cost of intervention ($US, millions)	Production losses averted ($US, millions)	DALY averted	Cost per DALY averted ($US)	Net cost per DALY averted ($US)
20 % subsidy	SES1–2	0·6	1·9	1648	354	−783
50 % subsidy		2·3	4·4	3918	587	−538
80 % subsidy		5·0	6·3	5801	866	−214
75 g/d for free		10·5	14·6	14 733	714	−276
20 % subsidy	SES1–4	1·2	3·5	2926	426	−759
50 % subsidy		4·7	8·1	6946	675	−498
80 % subsidy		10·0	11·6	10 256	977	−152
75 g/d for free		19·4	26·8	25 833	751	−286
20 % subsidy	SES1–6	2·1	5·2	4097	506	−773
50 % subsidy		7·5	12·4	9722	773	−498
80 % subsidy		15·7	17·6	14 348	1098	−129
75 g/d for free		28·4	40·3	35 735	795	−332
20 % subsidy	SES1–8	3·2	7·5	5480	590	−781
50 % subsidy		11·5	17·7	12 966	888	−474
80 % subsidy		23·9	25·1	19 050	1254	−65
75 g/d for free		38·3	55·2	45 571	841	−371

SES, socio-economic stratum (wealth index decile); DALY, disability-adjusted life year. The table shows the cost, effectiveness and cost-effectiveness of selected price-based interventions as well as of an intervention that provided all 6–23-month-old children of current and potential buyer households with the additional daily dose of 75 g.

### Cost-effectiveness of subsidies

Table 7 gives an overview of the results of our cost-effectiveness estimations. The costs of the interventions differed substantially between current buyers and potential buyers. While the price subsidies for potential buyers were limited to the additional quantity of FPCF bought because of the intervention, the price subsidies for current buyers also included the amount of FPCF that would have been bought anyway. This was a windfall gain for the household but an ineffective cost for the provider as it did not increase the consumption of FPCF (see online supplementary material, Section 7, Fig. S5).

The costs per DALY averted ranged from $US 354/DALY (20 % price subsidy for SES1–2) to $US 1254/DALY (80 % price subsidy for SES1–8). Applying the cost-effectiveness thresholds recommended by the WHO^(^ ^29^ ^)^, all the interventions appear highly cost-effective. The recommendations define an intervention as highly cost-effective if it meets a threshold per DALY averted of one times the annual gross domestic product per capita, which amounted to $US 1429 in Pakistan in 2015^(^ ^30^ ^)^. If taking a social perspective, which also considers the production losses averted, all interventions appear cost-saving as they not only averted DALY but also saved net social costs.

Interventions targeted at poorest household deciles were most cost-effective because: (i) the prevalence of IDA and VAD was higher; (ii) the share of non-buyers was slightly lower, making the interventions more effective; and (iii) the share of current buyers was lower, leading to lower ineffective intervention costs.

## Discussion

### Summary and interpretation of results

We estimated the cost-effectiveness of price subsidies on FPCF for the reduction of iodine, iron and vitamin A deficiencies in 6–23-month-old Pakistani children. For this purpose, we adapted a previous model of the societal costs of micronutrient deficiencies in Pakistan^(^ ^17^ ^)^ to the districts of Faisalabad and Hyderabad, and carried out a survey including a hypothetical marketing experiment among 1764 households. We then estimated the net cost per DALY averted of different price subsidies covering different socio-economic strata. Basing our calculation on the current MND status in Pakistan, we account for other fortification and supplementation programmes that are already in place.

We found that interventions targeting the poorest households are more cost-effective than interventions with a more extensive coverage because of the higher prevalence of MND, the lower levels of current consumption and the higher price elasticity of demand in low SES. From a societal perspective the most cost-effective intervention is a 20 % subsidy for the poorest 20 % of the population, with a net saving of $US 783 million per DALY averted.

While we found that interventions using FPCF could be effective in reducing the social cost of MND, even the most encompassing intervention could not eradicate IoD, IDA or VAD. Providing all 6–23-month-old Pakistani children with 75 g of FPCF daily could reduce the DALY due to IoD, IDA and VAD by 41·8 %. However, an 80 % subsidy for all households could avert only 12·0 % of DALY due to IoD, IDA and VAD. The main reason for this difference was that one in three households would not buy FPCF even at an 80 % discount (share of non-buyers, compare with Fig. 5). In comparison, the share of households who would not feed FPCF if they were for free was small with 3·5 % overall (6·2 % of current non-buyers). Price-based interventions with accompanying product information thus seem to be an effective instrument for demand creation but only in the case of sufficiently high price discounts of over 80 %.

### Comparison with previous studies

Comparing our results with those of other studies is rather difficult, due enormous differences in programme structures, delivery systems and other country-specific factors. Fiedler *et al.* ^(^ ^31^ ^)^ revealed a substantial variation in the estimated costs of fortification programmes and concluded that these costs are severely underestimated by a majority of the studies. A follow-up study by Fiedler and Macdonald^(^ ^32^ ^)^ assessed the intervention costs for four different food vehicles and forty-eight high-priority countries, including three interventions in Pakistan. Their results were slightly lower than those of our most cost-effective intervention, with estimated cost-effectiveness ratios as follows: vitamin A fortification of sugar at $US 616 per DALY averted; vitamin A fortification of vegetable oil at $US 289 per DALY averted; and wheat flour fortification with iron, folic acid and vitamin B_12_ at $US 341 per DALY averted.

### Possible under- and overestimation of cost-effectiveness

Our results may underestimate the cost-effectiveness of interventions for two reasons. First, FPCF provide children with macronutrients and not only with micronutrients. While the considerable cost for these macronutrients is considered in our analysis, their effects are not. Dewey and Adu-Afarwuah^(^ ^33^ ^)^ have shown that interventions providing additional energy positively affect growth, while interventions relying purely on fortification do not. Second, we included only iodine, iron and vitamin A in our calculations, while there are a number of other micronutrients that are contained in the fortified packaged infant cereals currently available in Pakistan. We excluded these other micronutrients and functional health outcomes and morbidity, because the systematic reviews on the effectiveness of complementary feeding interventions did not find any evidence for other micronutrients, while the evidence regarding functional health outcomes and morbidity was scarce and inconclusive^(^ ^18^ ^)^. However, this may not only be due to the absence of effects in published studies, but also due to a lack of reliable clinical studies.

On the other hand, we may overestimate the cost-effectiveness for the following reasons. First, we did not include programme implementation costs (including accompanying information campaigns), which may be substantial, especially for interventions targeting selected groups only. Second, some of the children included in the intervention already consumed large amounts of FPCF. Therefore, the effect of FPCF observed in the systematic review could be higher than real-world effects. Third, subsidized FPCF may substitute other foods in the diet of 6–23-month-old children. However, the same is true for the randomized trials regarding the effects of fortification on Hb and serum retinol levels, on which our study is based. While the effects on Hb and serum retinol levels thus take account of the likely substitution effects, the effects on iodine do not. However, as weaning foods (e.g. porridge, mashed fruits and vegetables, rice) are poor in native iodine, overestimation due to substitution effects might be rather small. Fourth, by using spot UIC as a biomarker of iodine intake we probably overestimate the prevalence of IoD, due to high day-to-day within-person variance of UIC^(^ ^34^ ^)^. Zimmermann *et al*.^(^ ^34^ ^)^ were the first to apply an external correction of the UIC distribution in order to estimate the prevalence of IoD. We addressed this possible overestimation with a univariate sensitivity analysis based on the findings of Zimmermann *et al*.^(^ ^34^ ^)^ (see online supplementary material, Section 8). Fifth, due to lack of data regarding the UIC values from 6–23-month-old children, we used the respective UIC values from SAC. There are no recent studies regarding complementary feeding practices in infants in Pakistan. Two studies^(^ ^35^ ^,^ ^36^ ^)^ using data from 2006–2007, as well as our conducted survey in 2015 (see online supplementary material, Section 6, Fig. S4), show that infants receive fortified formula in addition to breast milk. Fortified formulas often include iodine; as a result, iodine intake in infants may be higher than in SAC, despite the fact that SAC may consume other iodine-rich foods such as fish and iodized salt. Therefore, we likely overestimate the prevalence of IoD.

### Strengths and limitations

Our study has a number of strengths. A first strength is the combination of three distinct steps: a health economic model of the cost-consequences of MND in 6–23-month-old children, a systematic review of the effectiveness of FPCF in the reduction of IDA and VAD, and a household survey assessing the price sensitivity for FPCF in households with children in the relevant age group. A second strength is the application of health economic evaluation methodologies to the field of public health nutrition. This well-established methodological approach may contribute substantially to the evaluation and design of cost-effective nutritional interventions.

Our study also has a number of limitations. A first limitation is the hypothetical nature of the marketing experiment. Hypothetical questions assessing price sensitivity of demand are usually subject to bias^(^ ^37^ ^)^. However, our study design avoids the most important pitfalls of hypothetical questions: FPCF is a well-known and readily available product, and bought by many of our respondents. Those respondents not buying the product at the time of the survey were provided with a description of FPCF and shown an actual package to familiarize themselves with the product. The use of such existing, clearly specified products has been shown to improve the validity of purchasing intentions^(^ ^38^ ^)^. Obviously, a validation of our results in a real-world setting would be extremely useful, but it would require substantial financial and temporal resources. A real-world marketing experiment, however, could induce hoarding bias by encouraging respondents to buy higher quantity of FPCF than what they would normally buy. Hoarding bias should not be an issue in the hypothetical experiment. A second limitation is the uncertain accuracy of a number of assumptions in the health economic model. Assumptions about the long-term evolution of future wages and economic growth are particularly difficult to sustain. However, this type of modelling exercise is the only means of evaluating the cost-effectiveness of interventions aimed at reducing the lifetime consequences of MND in children^(^ ^39^ ^)^. A third limitation of our study is its short-term focus on increased buying of FPCF, which is only one element of a sustained change in complementary feeding practices. Other elements include the effective, repeated and coordinated provision of information as well as the regulation of marketing and promotion of these products^(^ ^13^ ^)^.

### Implementing interventions

Our results could be useful to policy makers constrained by limited budgets and given cost-effectiveness thresholds. For example, a budget of $US 5 million would be better spent on a 50 % subsidy for SES1–4 than on an 80 % subsidy for SES1–2, as it would be more cost-effective and avert a larger number of DALY (see Table 7). On the other side, a cost-effectiveness threshold of $US 1429/DALY (equal to the gross domestic product per capita) implies that the optimal intervention in terms of effectiveness would be the one providing FPCF for free to SES1–8 at a cost of $US 38·3 million. Policy makers would of course also need to consider the cost of implementing the interventions in their decisions.

Embedding the proposed interventions into a private–public partnership linked with public health policies and nutrition policies such as the Scaling-Up Nutrition Initiative^(^ ^40^ ^,^ ^41^ ^)^ would be key for feasibility and sustainability. A large-scale evaluation by GAIN has shown that such partnerships with the private sector are required to reach target consumers and drive demand for child nutrition products^(^ ^14^ ^)^. To ensure the maximum effectiveness of complementary feeding interventions, they should be ideally implemented in partnership with the health sector along with nutritional education. This education should cover the full range of child feeding practices from exclusive breast-feeding to initiating complementary infant food up to completely integrating the child into the family’s dietary routine^(^ ^42^ ^,^ ^43^ ^)^. Implementing the proposed interventions through such partnerships could substantially reduce the huge social and economic burden of child undernutrition in Pakistan.

## Footnotes

* Note that a log-linear model for potential buyer households cannot be estimated because their current demand is zero, as is the hypothetical demand at the first drawn discount for some of the households.

* Excluded observations received significantly less advice regarding child feeding and were less aware of VAD being harmful during early childhood.

## Supplementary material

For supplementary material accompanying this paper visit https://doi.org/10.1017/S1368980018001660. click here to view supplementary material
