# Supplementary material for: Reducing micronutrient deficiencies in Pakistani children: are subsidies on fortified complementary foods cost-effective?
Source: Public Health Nutr. 2018 Jul 18;21(15):2893–906. doi: 10.1017/S1368980018001660 (PMC6149007; doi:10.1017/S1368980018001660)
Supplement: Supplementary file 1 [file S1368980018001660sup001.docx]

**Supplementary material to
“Reducing Micronutrient Deficiencies in Pakistani Children: Are Subsidies on Fortified Complementary Foods Cost-Effective?”**

Content

[1. Health and Costs Consequences of examined MNDs 2](#_Toc507166682)

[2. Questionnaire 4](#_Toc507166683)

[3. Ex-post correction applied to hypothetical bias 27](#_Toc507166684)

[4. Robustness of estimated marginal demand effects 28](#_Toc507166685)

[5. Illustrative examples of cost-effectiveness calculations 30](#_Toc507166686)

[6. Results of the market survey 31](#_Toc507166687)

[7. Decomposition of effect of subsidies 32](#_Toc507166688)

[8. Sensitivity analyses 33](#_Toc507166689)

[References 37](#_Toc507166690)

1. Health and Costs Consequences of examined MNDs

The attribution of the health consequences to the examined MNDs is drawn from systematic reviews and primary supplementation trials in pre-school children. This previously applied approach ^(1)^ assumes that the supplements provided in these trials fill the necessary intake of micronutrients in this age-group. The health consequences are therefore measured as the difference in the examined health outcome between the intervention and control groups. Table S1 provides an overview of the concrete input parameters used to measure the health consequences of iodine, iron and Vitamin A deficiencies. Due to data limitations, the evidence regarding IoD was based on school-aged children. Table S2 provides an overview of the cost consequences of the adverse health effects due to iodine, iron and Vitamin A deficiencies.

Table S1: Health consequences of iodine, iron and Vitamin A deficiency

| **Micronutrient deficiency** | **Health Consequence** | **Effect Size (95% CI)** | **Persistent effect** | **Source** |
| --- | --- | --- | --- | --- |
| **Iodine deficiency  (IoD)** | Cognitive impairment:  Mild IoD 🡪 borderline and mild cognitive impairment  Moderate and Severe IoD 🡪 borderline, mild and moderate cognitive impairment | Mild IoD: total loss of 2.25 IQ points in school-age-children  Moderate IoD: total loss of 5.25 IQ points in school-age-children  Severe IoD: total loss of 20.25 IQ points in school-age-children and adults | Partially | ^(2)^;^(3)^ SR |
| **Iron  deficiency anemia  (IDA)** | Share of anemia caused by iron deficiency | 60% |  | ^(4)^ SR; ^(5)^ |
|  | Moderate IDA 🡪 borderline cognitive impairment  Severe IDA 🡪 mild cognitive impairment | Moderate and severe IDA: IQ in adults reduced by 9 IQ points | Yes | ^(6)^; ^(7)^; ^(8)^ GBD |
|  | Impaired physical activity | Mild to severe IDA: All children affected | No | ^(7)^ |
|  | All-cause mortality | Severe IDA: RR 2.19 | Yes | ^(9)^ SR |
| **Vitamin A deficiency (VAD)** | Xerophthalmia:  Bitot‘s spot | RR of 2.22 (1.64, 3.03) | No | ^(10)^ SR |
|  | Xerophthalmia:  Night blindness | RR of 3.13 (2.0, 4.76) | No |  |
|  | Diarrhea | RR of 1.18 (1.15, 1.22) | No |  |
|  | Measles | RR of 2.00 (1.49, 2.70) | No |  |
|  | All-cause mortality | RR of 1.32 (1.20, 1.45) | Yes |  |

GBD=Global Burden of Diseases study; RR=relative risk; SR=systematic review.

Source: Wieser et al. 2017 ^(2)^

Table S2: Cost consequences of adverse health effects

| **Health consequence** | **Cost category** | **Type and size of effect** | **Source** |
| --- | --- | --- | --- |
| Cognitive impairment | Production losses (future) | 1 SD reduction of the cognitive score leads to a reduction of adult wage by 8% | ^(11)^ |
|  | DALYs | Borderline intellectual functioning: DW 0.011  Mild intellectual disability: DW 0.043  Moderate intellectual disability: DW 0.1 | ^(8)^ GBD |
| Impaired physical activity | DALYs | Mild anemia: DW 0.004  Moderate anemia: DW 0.052  Severe anemia: DW 0.149 | ^(8)^ GBD |
| Bitot’s spot | DALYs | Moderate distance vision impairment: DW 0.031 | ^(8)^ GBD |
| Night blindness | DALYs | Moderate distance vision impairment: DW 0.031 | ^(8)^ GBD |
| Diarrhea | DALYs | Moderate diarrhea: DW 0.188 | ^(8)^ GBD |
| Measles | DALYs | Severe infectious disease: DW 0.133 | ^(8)^ GBD |

DW=disability weight; GBD=Global Burden of Diseases study.

Source: Wieser et al. 2017 ^(2)^

1. Questionnaire

To the best of our knowledge, there are iodized salt programs in place in Pakistan, despite the lack of published data to support their efficacy. **Section A3: Purchase & Consumption**

**A301** **What has [REFERENT CHILD] consumed yesterday and during the last night?**
OPEN QUESTION – DO NOT READ THE LIST
CIRCLE THE CONSUMED FOOD ITEMS CODES.
IN CASE OF EXCLUSIVE BREASTFEEDING GO TO SECTION 7

| **Table A31** | | | | |
| --- | --- | --- | --- | --- |
|  | **Food Codes** | **Yes** | **NO** | **DON’T KNOW** |
| Breast milk | 31 | 1 | 2 | 98 |
| Powdered milk | 32 | 1 | 2 | 98 |
| Fresh milk | 33 | 1 | 2 | 98 |
| Packaged infant cereals (powders like Cerelac) | 34 | 1 | 2 | 98 |
| Other packaged infant foods (like Gerber, Heinz) | 35 | 1 | 2 | 98 |
| Porridge, bread, rice, noodles, or other foods made from grains | 1 | 1 | 2 | 98 |
| Pumpkin, carrots, squash, or sweet potatoes that are yellow or orange inside | 2 | 1 | 2 | 98 |
| White potatoes, white yams, manioc, cassava, or any other foods made from roots | 3 | 1 | 2 | 98 |
| Any dark green leafy vegetables | 4 | 1 | 2 | 98 |
| Ripe mangoes, ripe papayas, or (insert other local vitamin A-rich fruits) | 5 | 1 | 2 | 98 |
| Any other fruits or vegetables | 6 | 1 | 2 | 98 |
| Liver, kidney, heart, or other organ meats | 7 | 1 | 2 | 98 |
| Any meat, such as beef, pork, lamb, goat, chicken, or duck | 8 | 1 | 2 | 98 |
| Eggs | 9 | 1 | 2 | 98 |
| Fresh or dried fish, shellfish, or seafood | 10 | 1 | 2 | 98 |
| Any foods made from beans, peas, lentils, nuts, or seeds | 11 | 1 | 2 | 98 |
| Cheese, yogurt, or other milk products | 12 | 1 | 2 | 98 |
| Any oil, fats, or butter, or foods made with any of these | 13 | 1 | 2 | 98 |
| Any sugary foods such as chocolates, sweets, candies, pastries, cakes, or biscuits | 14 | 1 | 2 | 98 |
| Condiments for flavor, such as chilies, spices, herbs, or fish powder | 15 | 1 | 2 | 98 |
| Grubs, snails, or insects | 16 | 1 | 2 | 98 |
| Goods made with red palm oil, red palm nut, or red palm nut pulp sauce | 17 | 1 | 2 | 98 |
| Others | 18 | 1 | 2 | 98 |
| Specify others (text) |  | | | |
| Don’t know / Don’t remember | 98 |  | | |

**A302 How many times did [REFERENT CHILD] eat foods, which are meals and snacks other than liquids, yesterday or at night?**

| number of times |  |
| --- | --- |
| Did not eat any thing | 77 |
| Don’t know / no answer | 98 |

**A303 Did you use salt to cook the food eaten by [referent child]?**

| Yes | 1 | - **GO TO A304** |
| --- | --- | --- |
| No | 2 | - **GO TO A310** |
| Don’t know / no answer | 98 |  |

**A304 What kind of salt did you use?**

IF POSSIBLE, ASK THE RESPONDENT TO SHOW YOU THE SALT

| Iodized | 1 |
| --- | --- |
| Not iodized | 2 |
| No salt at home | 3 |
| Don’t know / no answer | 98 |

**A310 Has [REFERENT CHILD] consumed packaged infant cereal in the last 3 days?**

| Yes | 1 | - **GO TO A311** |
| --- | --- | --- |
| No | 2 | - **GO TO A320** |
| Don’t know / no answer | 98 |  |

| **CLASSIFY AS:** | |
| --- | --- |
| If [REF CHILD] has consumed packaged infant cereal in last 3 days | **CURRENT BUYER** |
| Else | **NON BUYER** |

**A311 Which brands of packaged infant cereals did you serve to [REFERENT CHILD]?**RECORD IN COLUMN A311 OF TABLE A32

| **Table A32** | | | | | | | |
| --- | --- | --- | --- | --- | --- | --- | --- |
| **A311** | **A312** | **A313** | | | | | |
| **Brand name** | **MOST OFTEN CONSUMED PACKAGED INFANT CEREAL** | **Pack Size (ml or gram)** | | | | | |
|  |  | **RECORD PACK SIZE IN GRAMS (IF CAN NOT BE ASCERTAINED RECORD 9898)** | | | | **NOT AVAILABLE AT HOME CURRENTLY** | **REFUSED TO SHOW** |
|  | 1 |  |  |  |  | 1 | 2 |
|  | 2 |  |  |  |  | 1 | 2 |
|  | 3 |  |  |  |  | 1 | 2 |
|  | 4 |  |  |  |  | 1 | 2 |
|  | 5 |  |  |  |  | 1 | 2 |

**A312** **Now please tell me out of ____________ (MENTION BRANDS CODED IN A311 ONE BY ONE) which cereal does [REFERENT CHILD] consume the most?**

RECORD IN COLUMN A314 OF TABLE A32 – SINGLE CODING ONLY

INSTRUCTION TO INTERVIEWER: PLEASE ASK THE RESPONDENT TO SHOW THE PACKAGE AND VERIFY REPORTED PACK SIZE.

**A313 What size of _______________ (MENTION BRANDS CODED in A311 ONE BY ONE) did you buy?**

RECORD THE PACK SIZE IN GRAMS IN COLUMN A313 OF TABLE A32

INSTRUCTION TO INTERVIEWER: PLEASE ASK RESPONDENT TO BRING THE SPOON THEY NORMALLY USE TO POUR PACKAGED INFANT CEREAL WHILE PREPARING FOR CHILD

ALSO, TAKE OUT THE SEMOLINA PACKET

**A314 I have with me some semolina. Please tell me with the help of this spoon, how much quantity of packaged infant cereal you generally use when preparing one serving for_____________ (REFERENT CHILD)?**

ASK THE RESPONDENT TO PUT THE SEMOLINA IN SPOON.

MEASURE THE QUANTITY USING STANDARD BEAKER AND RECORD BELOW

| **QUANTITY LEVEL** |  |  |  |
| --- | --- | --- | --- |

**A315 Please tell me approx. how many servings does the child eat of this packaged infant cereal in a week?**

SINGLE CODING ONLY

| 6 times per day or more | 1 |
| --- | --- |
| 5 times per day | 2 |
| 4 times per day | 3 |
| 3 times per day | 4 |
| 2 times per day | 5 |
| 1 time per day | 6 |
| 5-6 times per week | 7 |
| 3-4 times per week | 8 |
| 1-2 times per week | 9 |
| Less often | 10 |
| During emergencies/ special occasions | 11 |

**A316 Please indicate the way this packaged infant cereal is generally prepared.**

SINGLE CODING ONLY

| Mixed with normal water | 1 |
| --- | --- |
| Mixed with warm water | 2 |
| Mixed with hot water | 3 |
| Mixed with normal milk | 4 |
| Mixed with warm milk | 5 |
| Mixed with hot milk | 6 |
| Any other (please specify) | |

**A317 How long back did you buy this packaged infant cereal?**

SINGLE CODING ONLY

| In last 1 week | 1 |
| --- | --- |
| In last 2 weeks but not in last 1 week | 2 |
| In last 1 month but not in last 2 weeks | 3 |
| In last 2 months but not in last 1 month | 4 |
| In last 6 months but not in last 2 months | 5 |
| Before last 6 months | 6 |

**A318 From which place do you purchase this packaged infant cereal most often?**

SINGLE CODING POSSIBLE

| General Store | 1 |
| --- | --- |
| Departmental Store | 2 |
| Super Store | 3 |
| Convenience Store | 4 |
| Pharmacy | 5 |
| Doctor | 6 |
| Bakery | 7 |
| Market | 8 |
| Others (Please specify) | |

**A319 Thinking about the total packaged infant cereal consumed in your household in last 1 week, please divide it among members in your household who consume packaged infant cereal as per their share in total quantity consumed.**

RECORD FOR EACH MEMBER CODED IN A314.

|  | **% Share** |
| --- | --- |
| Kids between 6-23 months |  |
| Kids between 2-5 years |  |
| Kids between 6-12 years |  |
| Kids between 13-17 years |  |
| Adults between 18-25 years |  |
| Adults between 26-40 years |  |
| Adults above 40 years |  |
|  | 100% |
| - **GO TO A328** | |

ASK A320-A326 IF PACKAGED INFANT CEREAL NOT CODED IN PREVIOUS SECTION

(A302 =”No”; PACKAGED INFANT CEREAL NOT SERVED IN PAST 3 DAYS)

**A320 Please tell me which brands of packaged infant cereals are you aware of?**DO NOT EXPOSE THE LIST. WRITE BRANDS IN 2^ND^ COLUMN OF TABLE A3.3
MULTIPLE CODING POSSIBLE

CODE 999 IN GRID IN A321.1 AND A321.1 IF RESPONDENT IS FAMILIAR WITH BRAND NAME BUT DOES NOT KNOW PRICE OR PACK SIZE.

| **Table A3.3** | | | | |
| --- | --- | --- | --- | --- |
| **A320.0** | | | **A321.1** | **A321.2** |
| **DO NOT EXPOSE THE LIST**  **DO NOT PROMPT** | | | **PRICE (in Rs.)** | **PACK SIZE**  **(in gm)** |
| Cerelac | Cerelac | 1 |  |  |
| other 1 |  | 2 |  |  |
| other 2 |  | 3 |  |  |
| other 3 |  | 4 |  |  |
| Not aware/ Cannot recall |  | 98 |  |  |

**A321 At which price is a pack of packaged infant cereal is available in market? Please specify the pack size as well (in gm).**

WRITE PRICE IN COLUMN A321.1 OF TABLE A3.3 AND PACK SIZE IN COLUMN A321.2.

**A322 Have you ever served packaged infant cereals to [REFERENT CHILD]?**

SINGLE CODING ONLY

| Yes | 1 | - **GO TO A323** |
| --- | --- | --- |
| No | 2 | - **GO TO A326** |
| Don’t know /  no answer | 98 |  |

**ASK A323 IF ‘1’ CODED IN A322**

**A323 Have you ever bought packaged infant cereals for [REFERENT CHILD]? SINGLE CODING ONLY**

| Yes | 1 | - **GO TO A324** |
| --- | --- | --- |
| No | 2 | - **GO TO A325** |
| Don’t know / no answer | 98 |  |

**A324 Could you please tell me why you do not give packaged infant cereals to [REFERENT CHILD] more often?**

MULTIPLE CODING POSSIBLE.

| I cannot afford packaged infant cereal | 1 | **GO TO A327** |
| --- | --- | --- |
| It is too expensive as compared to homemade food | 2 |  |
| There is no small pack to try it and if my child does not eat it, it will be a waste of money | 3 |  |
| It’s not healthy for my child | 4 |  |
| My child doesn’t like it | 5 |  |
| My child is too young for it | 6 |  |
| My child is too old for it | 7 |  |
| My child gets digestive problem after eating it | 8 |  |
| Homemade food has much more variety and child requires variety | 9 |  |
| It does not provide fat | 10 |  |
| My mother-in-law/ other family members/ friends are against it | 11 |  |
| The doctor advised me not to feed packaged food to my child | 12 |  |
| Do not have knowledge about all benefits it provides | 13 |  |
| It cannot be prepared and kept for a few hours like home food, it has to be eaten immediately | 14 |  |
| It is raw/ it is not made by boiling or cooking the food | 15 |  |
| It may contain preservatives which are harmful | 16 |  |
| The child will not develop habit of eating homemade food for later in life | 17 |  |
| It is very sticky and sticks into the mouth of the child | 18 |  |
| I feel guilty when I give packaged food to my child | 19 |  |
| Not available in the places where I go for shopping | 20 |  |
| Any other (please specify) | |  |
| Any other (please specify) | |  |

ASK A325 IF ‘2’ OR ‘98’ CODED IN A323

**A325 You said that you have never bought packaged infant cereals for [REFERENT CHILD]. May I know the reason why not?**

MULTIPLE CODING POSSIBLE

| I cannot afford packaged infant cereal | 1 | **GO TO A327** |
| --- | --- | --- |
| It is too expensive as compared to homemade food | 2 |  |
| There is no small pack to try it and if my child does not eat it, it will be a waste of money | 3 |  |
| It’s not healthy for my child | 4 |  |
| My child doesn’t like it | 5 |  |
| My child is too young for it | 6 |  |
| My child is too old for it | 7 |  |
| My child gets digestive problem after eating it | 8 |  |
| Homemade food has much more variety and child requires variety | 9 |  |
| It does not provide fat | 10 |  |
| My mother-in-law/ other family members/ friends are against it | 11 |  |
| The doctor advised me not to feed packaged food to my child | 12 |  |
| Do not have knowledge about all benefits it provides | 13 |  |
| It cannot be prepared and kept for a few hours like home food, it has to be eaten immediately | 14 |  |
| It is raw/ it is not made by boiling or cooking the food | 15 |  |
| It may contain preservatives which are harmful | 16 |  |
| The child will not develop habit of eating homemade food for later in life | 17 |  |
| It is very sticky and sticks into the mouth of the child | 18 |  |
| I feel guilty when I give packaged food to my child | 19 |  |
| Not available in the places where I go for shopping | 20 |  |
| Any other (please specify) |  |  |
| Any other (please specify) |  |  |

**ASK A326 IF ‘2’ CODED IN A322**

**A326 You said that you have never served packaged infant cereals to [REFERENT CHILD]. May I know the reason why not?**

MULTIPLE CODING POSSIBLE

| I cannot afford packaged infant cereal | 1 |
| --- | --- |
| It is too expensive as compared to homemade food | 2 |
| There is no small pack to try it and if my child does not eat it, it will be a waste of money | 3 |
| It’s not healthy for my child | 4 |
| My child doesn’t like it | 5 |
| My child is too young for it | 6 |
| My child is too old for it | 7 |
| My child gets digestive problem after eating it | 8 |
| Homemade food has much more variety and child requires variety | 9 |
| It does not provide fat | 10 |
| My mother-in-law/ other family members/ friends are against it | 11 |
| The doctor advised me not to feed packaged food to my child | 12 |
| Do not have knowledge about all benefits it provides | 13 |
| It cannot be prepared and kept for a few hours like home food, it has to be eaten immediately | 14 |
| It is raw/ it is not made by boiling or cooking the food | 15 |
| It may contain preservatives which are harmful | 16 |
| The child will not develop habit of eating homemade food for later in life | 17 |
| It is very sticky and sticks into the mouth of the child | 18 |
| I feel guilty when I give packaged food to my child | 19 |
| Not available in the places where I go for shopping | 20 |
| Any other (please specify) | |
| Any other (please specify) | |

**A327 Would you buy packaged infant cereals if you could afford it?**

SINGLE CODING ONLY

| Yes | 1 | - **GO TO A328** |
| --- | --- | --- |
| No | 2 |  |
| Don’t know /   no answer | 98 |  |

ASK ALL

**A328 Please tell me does your household receive packaged infant cereals from social programs?**

SINGLE CODING ONLY

*There are various food related social programs run by government or any social organization. Under these programs, food is provided to a household directly or indirectly generally either at no cost or at subsidized cost. Some examples of such programs are ration programs, school feeding programs, cash transfers for food etc.*

| Yes | 1 | **🡪 GO TO A329** |
| --- | --- | --- |
| No | 2 | **🡪 GO TO A400** |
| Don’t know / no answer | 98 |  |

ASK A329 IF ‘1’/’YES’ CODED IN A328

**A329 From which program did you receive the packaged infant cereals you gave to [REFERENT CHILD]?**

| Name of programme |  |
| --- | --- |

**Section A4: Price sensitivity**

**A400** **SELECT APPROACH 1 FOR CURRENT BUYERS AND APPROACH 2 FOR NON BUYERS**

|  | **A400** |  |
| --- | --- | --- |
| **Approach 1 if CURRENT buyer** | 1 | - **GO TO A401** |
| **Approach 2 if NON buyer** | 2 | - **GO TO A420** |

**APPROACH 1 - only current buyers**

**DETERMINE CURRENT BRAND**

**Brand (A304): _____________________**

**Pack Size (Pack size of brand coded in A305): ____________________**

**A401** **You mentioned that you serve [BRAND, PACK TYPE AND PACK SIZE] most often to your child. Please tell me at which price do you buy each pack of this brand, pack type and size?**
RECORD VERBATIM WITH LEADING ZEROES

| **PRICE OF MOST OFTEN USED PACK (in Rs.)** |  |  |  |  |
| --- | --- | --- | --- | --- |

**A402 How many packs of [BRAND, PACK TYPE AND PACK SIZE] do you buy in a usual shopping trip?**
RECORD VERBATIM WITH LEADING ZEORES

| **NUMBER OF PACKS PER SHOPPING TRIP** |  |  |
| --- | --- | --- |

**A403** **How often do you make such shopping trips?**
RECORD VERBATIM WITH LEADING ZEROES FOR NUMBER OF DAYS BETWEEN SHOPPING TRIPS

| **NUMBER OF DAYS BETWEEN SHOPPING TRIPS** |  |  |
| --- | --- | --- |

**A404** Instruction for Interviewer: **Record % DISCOUNT from RANDOMIZED LIST**

| **Discount in RANDOM LIST** | **SINGLE CODE** |
| --- | --- |
| 20% | 1 |
| 40% | 2 |
| 60% | 3 |
| 80% | 4 |

**A405** Instruction for Interviewer: **COMPUTE DISCOUNTED PRICE:**

| **DISCOUNT SELECTED** | **1** | **2** | **3** | **4** | **A405** |
| --- | --- | --- | --- | --- | --- |
| **Brand _________** | **DISCOUNT - 20%** | **DISCOUNT - 40%** | **DISCOUNT –**  **60%** | **DISCOUNT - 80%** | **DISCOUNTED PRICE** |
| **Price Paid per Pack (A401)** _________ | ___ 0.8 | ___ 0.6 | ___ 0.4 | ___ 0.2 |  |

| **EXAMPLE FOR COMPUTING DISCOUNTED PRICE:**  **Price paid per pack – Rs. 145**  **Discount Selected in A404 – ‘2’/ Discount 40%**  **DISCOUNTED PRICE = 145 x 0.6**  **= Rs. 87** | | | | | |
| --- | --- | --- | --- | --- | --- |
| **DISCOUNT SELECTED** | **1** | **2** | **3** | **4** |  |
|  | **DISCOUNT - 20%** | **DISCOUNT - 40%** | **DISCOUNT - 60%** | **DISCOUNT - 80%** | **DISCOUNTED PRICE** |
| **Price Paid per Pack (Q401)** **Rs. 145** | ___ 0.8 | X 0.6 | ___ X 0.4 | ___ X 0.2 | **Rs. 87** |

**ASK A406- A415 FOR DISCOUNTED PRICE**

**SAY:** We are interested in finding out how much packaged infant cereal you would buy at a lower price. This lower price would be a permanently lower price and not just a temporary promotion price. At this price, product is going to be regularly available in the market

**A406 Please give me your opinion on the value of money of this packaged infant cereal if one package of this was available at** **[DISCOUNTED PRICE]**? **SINGLE CODING ONLY**

| Excellent | 5 |
| --- | --- |
| Very good | 4 |
| Good | 3 |
| Fair | 2 |
| Poor | 1 |
| Don’t know **(DO NOT ASK)** | 98 |

**A407 Please tell me how willing you are to buy this packaged infant cereal in the future if one package of this was available at [DISCOUNTED PRICE]?**

SINGLE CODING ONLY

| Will definitely buy | 5 | **🡪 GO TO A408** |
| --- | --- | --- |
| Will probably buy | 4 |  |
| May or may not buy | 3 |  |
| Will probably not buy | 2 |  |
| Will definitely not buy | 1 | **🡪 GO TO A501** |
| Don’t know **(DO NOT ASK)** | 98 | **🡪 GO TO A408** |

**ASK A408 IF CODED 2 / 3 / 4 / 5 / 98 IN A407**

**A408 How many packs of [BRAND NAME, PACK TYPE AND PACK SIZE] would you buy in a usual shopping trip if the price of a package was** **[DISCOUNTED PRICE]**?

RECORD VERBATIM WITH LEADING ZEROES

| **NUMBER OF PACKS PER SHOPPING TRIP** |  |  |
| --- | --- | --- |

**A409 How often would you make such shopping trip?**

RECORD VERBATIM WITH LEADING ZEROES FOR NUMBER OF DAYS BETWEEN SHOPPING TRIPS

| **NUMBER OF DAYS BETWEEN SHOPPING TRIPS** |  |  |
| --- | --- | --- |

**A410 Will you serve more packaged infant cereal as a result of this lower price or will consumption remain the same?** SINGLE CODING ONLY

| Yes, will serve more packaged infant cereal | 1 | **🡪 GO TO A411** |
| --- | --- | --- |
| No, will NOT serve more packaged infant cereal/ Consumption will remain the same | 2 | **🡪 GO TO A501** |
| Don’t know **(DO NOT ASK)** | 98 | **🡪 GO TO A411** |

**ASK A411 IF CODED 1 OR 98 IN A410**

**A411 If you will serve more packaged infant cereal than you currently do, who would consume the ADDITIONAL packaged infant cereal?**

SINGLE CODING ONLY

| Equally distributed among adults and children | 1 | **🡪 GO TO A412** |
| --- | --- | --- |
| Only adults | 2 | **🡪 GO TO A501** |
| Mainly adults | 3 |  |
| Mainly children | 4 | **🡪 GO TO A412** |
| Only child/ children | 5 |  |
| Don’t know **(DO NOT ASK)** | 98 | **🡪 GO TO A501** |

**ASK A412 IF CODED 1 / 4 / 5 IN A411**

**A412** **How will the ADDITIONAL packaged infant cereal be distributed among the children?**SINGLE CODING ONLY
IF ONLY 1 CHILD IN THE FAMILY, AUTOCODE 2

| Equally distributed among all children | 1 | **🡪 GO TO A413** |
| --- | --- | --- |
| Only **[REFERENT CHILD]** | 2 |  |
| Mainly **[REFERENT CHILD]** | 3 |  |
| Mainly the other children | 4 |  |
| Only the other children | 5 | **🡪 GO TO A501** |
| Don’t know **(DO NOT ASK)** | 98 | **🡪 GO TO A413** |

**ASK A413 IF CODED 1 / 2 / 3 / 4 / 98 IN A412**

**A413** **Would you feed less of other food and drinks for** **[REFERENT CHILD]**?

SINGLE CODING ONLY

| Yes | 1 | **🡪 GO TO A414** |
| --- | --- | --- |
| No | 2 | **🡪 GO TO A501** |
| Don’t know **(DO NOT ASK)** | 98 |  |

**ASK A414 IF 1 / ‘YES’ CODED IN A413**

**A414** **What kind of food and drinks would you feed less to [REFERENT CHILD]?**

MULTIPLE CODING POSSIBLE

| **same as table A31** | | | | |
| --- | --- | --- | --- | --- |
|  | **Food Codes** | **Yes** | **NO** | **DON’T KNOW** |
| Breast milk | 31 | 1 | 2 | 98 |
| Powdered milk | 32 | 1 | 2 | 98 |
| Fresh milk | 33 | 1 | 2 | 98 |
| Packaged infant cereals (powders like Cerelac) | 34 | 1 | 2 | 98 |
| Other packaged infant foods (like Gerber, Heinz) | 35 | 1 | 2 | 98 |
| Porridge, bread, rice, noodles, or other foods made from grains | 1 | 1 | 2 | 98 |
| Pumpkin, carrots, squash, or sweet potatoes that are yellow or orange inside | 2 | 1 | 2 | 98 |
| White potatoes, white yams, manioc, cassava, or any other foods made from roots | 3 | 1 | 2 | 98 |
| Any dark green leafy vegetables | 4 | 1 | 2 | 98 |
| Ripe mangoes, ripe papayas, or (insert other local vitamin A-rich fruits) | 5 | 1 | 2 | 98 |
| Any other fruits or vegetables | 6 | 1 | 2 | 98 |
| Liver, kidney, heart, or other organ meats | 7 | 1 | 2 | 98 |
| Any meat, such as beef, pork, lamb, goat, chicken, or duck | 8 | 1 | 2 | 98 |
| Eggs | 9 | 1 | 2 | 98 |
| Fresh or dried fish, shellfish, or seafood | 10 | 1 | 2 | 98 |
| Any foods made from beans, peas, lentils, nuts, or seeds | 11 | 1 | 2 | 98 |
| Cheese, yogurt, or other milk products | 12 | 1 | 2 | 98 |
| Any oil, fats, or butter, or foods made with any of these | 13 | 1 | 2 | 98 |
| Any sugary foods such as chocolates, sweets, candies, pastries, cakes, or biscuits | 14 | 1 | 2 | 98 |
| Condiments for flavor, such as chilies, spices, herbs, or fish powder | 15 | 1 | 2 | 98 |
| Grubs, snails, or insects | 16 | 1 | 2 | 98 |
| Goods made with red palm oil, red palm nut, or red palm nut pulp sauce | 17 | 1 | 2 | 98 |
| Others (see table A3.1) | 18 | 1 | 2 | 98 |
| Don’t know | 98 |  | | |

**A415** **Please tell me whether you would buy less of other brands of packaged infant cereals that you buy regularly?
MULTIPLE CODING POSSIBLE**

| **Specifiy brand name** | **BRANDS CODED IN A303** |
| --- | --- |
|  | 1 |
|  | 2 |
|  | 3 |
|  | 4 |
|  | 5 |
| **Will not reduce for any brand** | 09 |
| **🡪 GO TO A501** | |

**APPROACH 2 - only non-buyers**

**SAY:** Many children suffer from micronutrient deficiencies and therefore cannot develop optimally. Suppose you were eligible for a program allowing you, as the mother of a child aged 6-23 months old, to buy packaged infant cereal at a reduced price. These infant cereals are a nutritious baby food containing added minerals and vitamins. They support the healthy growth and development of your child. The added minerals and vitamins may help protect your child from infections, eye problems, weak memory and ill health in general. Each pack of this special infant cereal for children 6-23 months of age would be sufficient to prepare 1 serving of cereals for **[REFERENT CHILD]**. The required daily amount of this infant cereal is 3 servings per day i.e. 3 packs per day.

Please note that this product is not a substitute for breastfeeding and that **[REFERENT CHILD]** will still require other food items along with this.

This special price would be a permanently lower price and not just a temporary promotion price. At this price, the product is going to be regularly available in the market.

According to the programme (child 6-23 months of age) should be fed with 3 packages per day until she / he reaches the age of 2 years. Please note that this packaged infant cereal is for children 6 months to less than 2 years old only and is not advisable to be served to others not entailed in this age range.

We are interested in finding out how much of this packaged infant cereal you would buy.

**EXPOSE CONCEPT CARD AND DEBRANDED PACKS**

**A420 Select START_PRICE _2 from RANDOM LIST.**

**START WITH THAT PRICE**

| **SINGLE CODING ONLY** | | | | |
| --- | --- | --- | --- | --- |
| **P1**  **Rs. 14** | **P2**  **Rs. 11** | **P3**  **Rs. 8** | **P4**  **Rs. 5** | **P5**  **Rs. 2** |
| 1 | 2 | 3 | 4 | 5 |

**A421** **Would you buy this packaged infant cereal if the price of 1 package was [MENTION PRICE]?**

SINGLE CODING ONLY

| Yes | 1 | **🡪 GO TO A423** |
| --- | --- | --- |
| No | 2 | **🡪 GO TO A422** |
| Don’t know **(DO NOT SHOW)** | 98 |  |

**A422** **Would you buy this packaged infant cereal if the price of 1 package was [NEXT LOWER PRICE]?**

If “NO” CONTINUE TO NEXT LOWER PRICE UNTIL LOWEST PRICE.

SINGLE CODING ONLY

| **CIRCLE THE STARTING PRICE** | 2 | 3 | 4 | 5 |  |
| --- | --- | --- | --- | --- | --- |
| **= NEXT LOWER PRICE TO START_PRICE_2** | **Rs. 11** | **Rs. 8** | **Rs. 5** | **Rs. 2** |  |
| **CIRCLE THE PRICE AT WHICH RESPONDENT SAID ‘YES’** | **2** | **3** | **4** | **5** | **None - 9** |
|  | **🡪 GO TO A423** | | | | **🡪 GO TO A432** |

**ASK A423 FOR PRICE FOR WHICH 1’ / YES CODED IN A421 OR A422 = PRICE_ACCEPT**

| **WRITE PRICE_ACCEPT INTO BOX:** |  |  |
| --- | --- | --- |

**A423** **Would you feed less of other food and drinks for [REFERENT CHILD]?** SINGLE CODING ONLY

| Yes | 1 | **🡪 GO TO A424** |
| --- | --- | --- |
| No | 2 | **🡪 GO TO A425** |
| Don’t know **(DO NOT SHOW)** | 98 |  |

**ASK A424 IF ‘1’ CODED IN A423**

**A424** **What kind of food and drinks would you feed less to [REFERENT CHILD]**? MULTIPLE CODING POSSIBLE

| **same as table A31** | | | | |
| --- | --- | --- | --- | --- |
|  | **Food Codes** | **Yes** | **NO** | **DON’T KNOW** |
| Breast milk | 31 | 1 | 2 | 98 |
| Powdered milk | 32 | 1 | 2 | 98 |
| Fresh milk | 33 | 1 | 2 | 98 |
| Packaged infant cereals (powders like Cerelac) | 34 | 1 | 2 | 98 |
| Other packaged infant foods (like Gerber, Heinz) | 35 | 1 | 2 | 98 |
| Porridge, bread, rice, noodles, or other foods made from grains | 1 | 1 | 2 | 98 |
| Pumpkin, carrots, squash, or sweet potatoes that are yellow or orange inside | 2 | 1 | 2 | 98 |
| White potatoes, white yams, manioc, cassava, or any other foods made from roots | 3 | 1 | 2 | 98 |
| Any dark green leafy vegetables | 4 | 1 | 2 | 98 |
| Ripe mangoes, ripe papayas, or (insert other local vitamin A-rich fruits) | 5 | 1 | 2 | 98 |
| Any other fruits or vegetables | 6 | 1 | 2 | 98 |
| Liver, kidney, heart, or other organ meats | 7 | 1 | 2 | 98 |
| Any meat, such as beef, pork, lamb, goat, chicken, or duck | 8 | 1 | 2 | 98 |
| Eggs | 9 | 1 | 2 | 98 |
| Fresh or dried fish, shellfish, or seafood | 10 | 1 | 2 | 98 |
| Any foods made from beans, peas, lentils, nuts, or seeds | 11 | 1 | 2 | 98 |
| Cheese, yogurt, or other milk products | 12 | 1 | 2 | 98 |
| Any oil, fats, or butter, or foods made with any of these | 13 | 1 | 2 | 98 |
| Any sugary foods such as chocolates, sweets, candies, pastries, cakes, or biscuits | 14 | 1 | 2 | 98 |
| Condiments for flavor, such as chilies, spices, herbs, or fish powder | 15 | 1 | 2 | 98 |
| Grubs, snails, or insects | 16 | 1 | 2 | 98 |
| Goods made with red palm oil, red palm nut, or red palm nut pulp sauce | 17 | 1 | 2 | 98 |
| Others (see table A3.1) | 18 | 1 | 2 | 98 |
| Don’t know | 98 |  | | |

**A425 Who in your household would consume the packaged infant cereal?**

SINGLE CODING ONLY

| Equally distributed among adults and children | 1 | **🡪 GO TO A426** |
| --- | --- | --- |
| Only adults | 2 | **🡪 GO TO A427** |
| Mainly adults | 3 |  |
| Mainly children | 4 | **🡪 GO TO A426** |
| Only child/ children | 5 |  |
| Don’t know **(DO NOT SHOW)** | 98 | **🡪 GO TO A427** |

**ASK A426 IF CODED 1 / 4 / 5 IN A425**

**A426** **How will the packaged infant cereal be distributed among the children?**
SINGLE CODING ONLY

**IF ONLY 1 CHILD IN THE FAMILY, AUTOCODE ‘2’**

| Equally distributed among all children | 1 |
| --- | --- |
| Only **[REFERENT CHILD]** | 2 |
| Mainly **[REFERENT CHILD]** | 3 |
| Mainly the other children | 4 |
| Only the other children | 5 |
| Don’t know **(DO NOT ASK)** | 98 |

**ASK ALL**

**A427 Please give me your opinion on the value of money of this packaged infant cereal if one package of this was available at (PRICE_BUY_ACCEPT)?** SINGLE CODING ONLY

| Excellent | 5 |
| --- | --- |
| Very good | 4 |
| Good | 3 |
| Fair | 2 |
| Poor | 1 |
| Don’t know **(DO NOT ASK)** | 98 |

**A428 Please tell me how willing are you to buy this packaged infant cereal in the future if one package of this was available at (PRICE_ACCEPT)?** SINGLE CODING ONLY

| Will definitely buy | 5 | **🡪 GO TO A429** |
| --- | --- | --- |
| Will probably buy | 4 |  |
| May or may not buy | 3 |  |
| Will probably not buy | 2 |  |
| Will definitely not buy | 1 | **🡪 GO TO A501** |
| Don’t know **(DO NOT ASK)** | 98 |  |

**ASK A429 IF CODED 5 / 4 / 3 / 2 IN A428**

**A429** **How many packs of [CONTROL PACK] would you buy in a week at PRICE_ACCEPT?**

RECORD VERBATIM WITH LEADING ZEROES

| **NUMBER OF PACKS PER WEEK** |  |  |
| --- | --- | --- |

**A430** **How many servings of [CONTROL PACK] would you give to [REFERENT CHILD] in a week?**RECORD VERBATIM WITH LEADING ZEROES

| **NUMBER OF SERVINGS PER WEEK** |  |  |
| --- | --- | --- |

**A431** **You said that you would buy _________ [NUMBER OF PACKS ACCORDING TO A429] for ___________ Rs. per week [NUMBER OF PACKS ACCORDING TO A429 MULTIPLIED PER PRICE_ACCEPT].
Is this true?**

| **Yes, true** | 1 | **🡪 GO TO A501** |
| --- | --- | --- |
| **No, false** | 2 | **🡪 GO BACK TO A429** |

| **EXAMPLE FOR COMPUTING AMOUNT SPEND PER WEEK ACCORDING TO PRICE_ACCEPT AND ANSWER A429:**   \| **PRICE_ACCEPT** \| **NUMBER OF PACKS BOUGHT PER WEEK (A429)** \| **AMOUNT SPENT PER WEEK ON PIC** \| \| --- \| --- \| --- \| \| **11** \| **6** \| **11 × 6 = 66** \| |
| --- | --- | --- | --- | --- | --- | --- |

**ASK A432 IF CODED 9 IN A422**

**A432** **Would you feed [CONTROL PACK] to [REFERENT CHILD] if you received [CONTROL PACK] for FREE? SINGLE CODING ONLY**

| Yes | 1 | **🡪 GO TO A501** |
| --- | --- | --- |
| No | 2 |  |
| Don’t know **(DO NOT SHOW)** | 98 |  |

**Section A5: Nutritional Knowledge and attitudes**

“Now I would like to ask you some questions on your knowledge about nutrition in general.”

**A501** **Did you receive any advice on which type of foods/ drinks you should give to your child?**

SINGLE CODING ONLY

| Yes | 1 | **🡪 GO TO A502** |
| --- | --- | --- |
| No | 2 | **🡪 GO TO A503** |
| Don't know | 98 |  |

**A502** **Do you remember who gave you the advice?**

MULTIPLE CODING POSSIBLE

| A paediatrician | 1 |
| --- | --- |
| Another type of health professional | 2 |
| A midwife | 3 |
| A relative (own mom, aunt, mother-in-law) or friend | 4 |
| Information from TV, radio, newspaper or other media | 5 |
| Others, please specify: | |
| Don't know/Don't remember  **(DON’T SHOW/ READ)** | 98 |

**A503 How long is it recommended that a woman breastfeeds her child?**

PROBE IF NECESSARY: **Until what age is it recommended that a mother continues breastfeeding?**

SINGLE CODING ONLY

| 6 months or less | 1 |
| --- | --- |
| 6–11 months | 2 |
| 12–23 months | 3 |
| 24 months and more | 4 |
| Other | 5 |
| Don’t know / no response | 98 |

**A504 At what age should babies start eating foods in addition to breastmilk?**

SINGLE CODING ONLY

| before 4 months of age | 1 |
| --- | --- |
| between 4-6 months of age | 2 |
| at 6 months of age | 3 |
| between 6-8 month of age | 4 |
| after 8 months of age | 5 |
| Don’t know / no response | 98 |

**A505 At what age did [REFERENT CHILD] start eating foods in addition to breastmilk?**

SINGLE CODING ONLY

| before 4 months of age | 1 |
| --- | --- |
| between 4-6 months of age | 2 |
| at 6 months of age | 3 |
| between 6-8 month of age | 4 |
| after 8 months of age | 5 |
| Don’t know / no response | 98 |

**A510 Have you heard about iron-deficiency anaemia?**

SINGLE CODING ONLY

| Yes | 1 | **🡪 GO TO A511** |
| --- | --- | --- |
| No | 2 | **🡪 GO TO A520** |
| Don’t know /  no answer | 98 |  |

ASK A511 IF CODED ‘1’ IN A510

**A511 Can you tell me how you can recognize someone who has anaemia?**

SINGLE CODING ONLY

| Less energy/weakness | 1 |
| --- | --- |
| Paleness/pallor | 2 |
| Spoon nails/bent nails | 3 |
| More likely to become sick | 4 |
| Other | 5 |
| Don’t know / no answer | 98 |

**A512 Are there any foods containing iron?**

SINGLE CODING ONLY

| Yes | 1 | **🡪 GO TO A513** |
| --- | --- | --- |
| No | 2 | **🡪 GO TO A514** |
| Don't know /no answer | 98 |  |

ASK A513 IF CODED ‘1’ IN A512

**A513 Do you know which foods and drinks are rich in iron?**

MULTIPLE CODING POSSIBLE

| Breast Milk | 1 |
| --- | --- |
| Cow milk / buffalo mild (packaged or fresh) | 2 |
| Infant formula | 3 |
| Packaged Powdered Milk | 4 |
| Packaged Infant Cereals | 5 |
| Bread/Roti | 6 |
| Rice | 7 |
| Maize | 8 |
| Pulses/Dal | 9 |
| Nuts | 10 |
| Vegetables and vegetable soups | 11 |
| Fruits/ Fruit Juices | 12 |
| Meat / Chicken / Fish | 13 |
| Egg | 14 |
| Milk products like jogurt, cheese, butter, cream, tea, coffee, shakes, ice cream etc | 15 |
| Oil | 16 |
| Packaged snacks/ foods like biscuits, chips, soft drinks, confectionary items, namkeens, milk additives likes Horlicks etc. | 17 |
| Any other | 18 |
| Don't know /no answer | 98 |

**A514 What are the health consequences of too little iron in foods and drinks?**

MULTIPLE CODING POSSIBLE

| Impact on eyesight | 1 |
| --- | --- |
| Anemia/ Blood deficiency | 2 |
| Lethargy/ Weakness | 3 |
| Skin Related problems like allergy, rashes | 4 |
| Hair related problems | 5 |
| Irritability | 6 |
| Problems in digestion | 7 |
| Impact on bones strength | 8 |
| Mental development | 9 |
| Others, please specify: | |
| Don't know/Don't remember | 98 |

**A520 Have you heard about vitamin A deficiency or lack of vitamin A?**

SINGLE CODING ONLY

| Yes | 1 | **🡪 GO TO A521** |
| --- | --- | --- |
| No | 2 | **🡪 GO TO A530** |
| Don’t know /  no answer | 98 |  |

**A521 How can one prevent a lack of vitamin A in the body?**

MULTIPLE CODING POSSIBLE

| Feed vitamin-A-rich foods | 1 |
| --- | --- |
| Feed foods fortified with vitamin A | 2 |
| Give vitamin A supplements | 3 |
| Other | 4 |
| Don’t know / no response | 98 |

**A530 Do you know what iodine deficiency is?
PROBE IF NECESSARY: Have you heard about iodine deficiency?**

SINGLE CODING ONLY

| Yes | 1 | **🡪 GO TO A531** |
| --- | --- | --- |
| No | 2 | **🡪 GO TO A540** |
| Don’t know /  no answer | 98 |  |

**A531 How can iodine deficiency be prevented?**

MULTIPLE CODING POSSIBLE

| Eat/prepare foods with iodized salt | 1 |
| --- | --- |
| Other | 2 |
| Don’t know /  no answer | 98 |

**ASK A540-A544 IF PACKAGED INFANT CEREAL CODED IN A310 (SERVED IN PAST 3 DAYS)**

**A540 Do you serve packaged infant cereals that contain added vitamins and minerals?**

SINGLE CODING ONLY

| Yes | 1 |
| --- | --- |
| No | 2 |
| Don't know/Don't remember | 98 |

**A541 Until what age do you plan to provide packaged infant cereals to your child?**

IF DON’T KNOW RECORD 98

| Maximum age of child (in years) |  |  |
| --- | --- | --- |

**A542 Have you received any advice on giving packaged infant cereals to [REFERENT CHILD]?**

SINGLE CODING ONLY

| Yes, I have received advice **NOT** to give packaged infant cereals to **[REFERENT CHILD]** | 1 | **🡪 GO TO A543** |
| --- | --- | --- |
| Yes, I have received advice to give packaged infant cereals to **[REFERENT CHILD]** | 2 |  |
| No, I have not received any advice | 3 | **🡪 GO TO SECTION 6** |
| Don't know/Don't remember **(DON’T ASK)** | 98 |  |

**A543 Do you remember who gave you the advice?**

MULTIPLE CODING POSSIBLE

| A paediatrician | 1 |
| --- | --- |
| Another type of health professional | 2 |
| A midwife | 3 |
| A relative (own mom, aunt, mother-in-law) or friend | 4 |
| Information from TV, radio, newspaper or other media | 5 |
| Others, please specify: | 6 |
| Don't know/Don't remember | 98 |

**A544 What was the age of [REFERENT CHILD] when the advice was given?**

RECORD IN MONTHS.

IF ADVICE WAS GIVEN DURING PREGNANCY RECORD “00”

| **Age of child (IN MONTHS) when advice was given** |  |  |
| --- | --- | --- |

- **GO TO Section 6**

**Section A6: Occurrence and treatment of illnesses**

**DIARRHEA:**

**Q601 When was REFERENT CHILD the last time sick with diarrhea (Having three or more loose or liquid stools per day)?**

SINGLE CODING ONLY

| currently | 1 | **🡪 GO TO Q602** |
| --- | --- | --- |
| during past 2 weeks | 2 |  |
| during past 3 months | 3 |  |
| Never | 4 | **🡪 GO TO Q605** |
| Don’t know | 98 |  |

**Q602 When REFERENT CHILD was last sick with diarrhea did you take him/ her to the doctor?**

SINGLE CODING ONLY

| Yes | 1 | **🡪 GO TO Q603** |
| --- | --- | --- |
| No | 2 | **🡪 GO TO Q605** |
| Don’t know **(DO NOT SHOW)** | 98 |  |

**Q603 How much did you spend for the treatment of diarrhea?**

CODE “O” IF NO EXPENDITURES

| Consultation and medical costs (in Rs.): |  |
| --- | --- |
| Medicne (in Rs.): |  |
| Investigation (in Rs.): |  |
| Hospitalization (if hospitalized in emergency or hospital for the treatment of diarrheoa ) (in Rs.): |  |
| Transportation (in Rs.): |  |

**Q604 How many days did you and youe spouse take off from work in order to care the child with diarrhea?**

CODE “O” IF NO DAYS OFF

| number of days off work by RESPONDENT due to care of child with diarrhea |  |
| --- | --- |
| number of days off work by SPOUSE due to care of child with diarrhea |  |

**ACUTE RESPIRATORY INFECTION (ARI):**

**Q605 When was REFERENT CHILD the last time sick with acute respiratory infection (ARI)?**

SINGLE CODING ONLY

| currently | 1 | **🡪 GO TO Q606** |
| --- | --- | --- |
| during past 2 weeks | 2 |  |
| during past 3 months | 3 |  |
| Never | 4 | **🡪 GO TO Section 7** |
| Don’t know | 98 |  |

**Q606 When REFERENT CHILD was last sick with ARI did you take him/ her to the doctor?**

SINGLE CODING ONLY

| Yes | 1 | **🡪 GO TO Q607** |
| --- | --- | --- |
| No | 2 | **🡪 GO TO Section 7** |
| Don’t know **(DO NOT SHOW)** | 98 |  |

**Q607** How much did you spend for the treatment of ARI (consultation and medication cost)?

CODE “O” IF NO EXPENDITURES

| Consultation and medical costs (in Rs.): |  |
| --- | --- |
| Medicne (in Rs.): |  |
| Investigation (in Rs.): |  |
| Hospitalization (if hospitalized in emergency or hospital for the treatment of ARI) (in Rs.): |  |
| Transportation (in Rs.): |  |

**Q608 How many days did you and your spouse take off from work in order to care the child with ARI?**

CODE “O” IF NO DAYS OFF

| number of days off work **by RESPONDENT** due to care of child with ARI |  |
| --- | --- |
| number of days off work **by** **SPOUSE** due to care of child with ARI |  |

Fig. S1: De-branded FPCFs


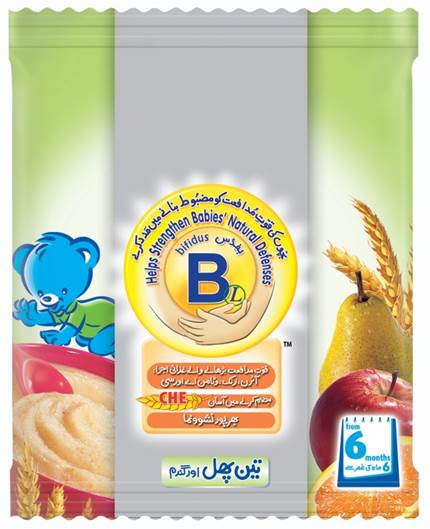

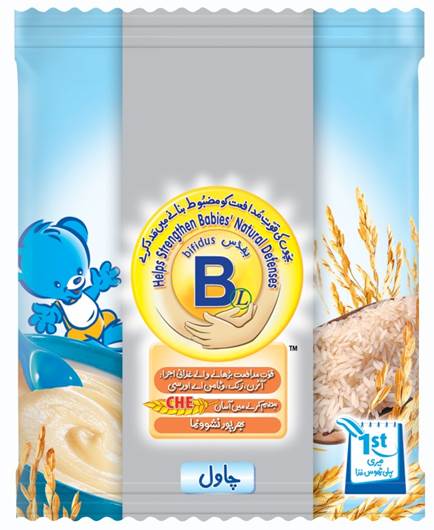

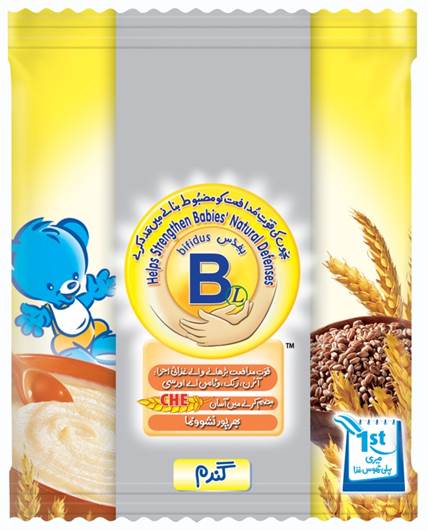


1. Ex-post correction applied to hypothetical bias

We addressed potential acquiescence and response bias, that may occur in hypothetical experiments, with the certainty scale methodology proposed by Blumenschein et al. ^(12)^. Respondents were additionally asked about their certainty related to the actual realization of their stated purchase of FPCF. Uncertain responders were excluded from the analysis. Such ex-post corrections have been shown to provide unbiased willingness-to-pay estimates that reflect actual behavior ^(13)^.

1. Robustness of estimated marginal demand effects

We tested the robustness of our baseline Fixed-Effects estimation results in two ways: First, we ~~Figure A.2 shows the estimated marginal demand effect by SES and their 95% CI for current and potential buyers for the baseline model applied in our study and for an alternative model. In contrast to the baseline model, the alternative model is not estimated in first difference but is~~estimated a semi-parametric F~~f~~ixed E~~e~~ffects model including 10 dummy variables for SES. Fig. S2 shows the results for both current and potential buyers. It displays the marginal demand effect by SES along with the 95% CI both for the baseline model applied in our study and for the semi-parametric approach. It can be seen that the variation in the demand effect across SES is modelled more precisely in the baseline model assuming constant effects compared to the ~~alternative~~ semi-parametric model.

Fig. S2: Comparing marginal effects between alternative and baseline model

Source: Authors’ calculation.

The dotted lines with the bullets show the baseline estimates and the dark grey area shows their 95% CI. The dotted lines with the crosses show the semi-parametric estimates and the light grey area shows their 95% CI.

Second, we estimated different limited dependent variable models; (1) Tobit Model, (2) Hurdle Model, (3) Heckman Model. These models take into account that demand is bounded by zero and that the change in demand is the result of two decision processes; The first decision is binary “whether or not” to respond to price discounts. For those who respond, the second decision is about “how much”, and therefore continuous. The continuous decision is modeled analogously to our baseline Fixed-Effects model. The binary decision is modeled as follows (except for the Tobit Model which assumes the two equations to be the same):

$${P(y>0)}_{i}=\alpha+ \beta\mathrm{SES}_{i}+\gamma\mathrm{SES}_{i}^{2}+\delta D_{i}+\theta\mathbf{X}_{i}+ \varepsilon_{i}$$

${P(y>0)}_{i}$is the probability that individual increases demand as result of the price discount (D$)$. This probability is expected to depend on wealth ($\mathrm{SES}_{i}$) and additional socio-economic variables**.** In particular the vector **X** contains dummy variables for the highest educational attainment, working status and the relationship of the decision maker to the child (mother, father, grandmother, aunt, uncle, other). It further contains the age of the child and whether or not the decision maker has received nutritional advice. Other indicators for health and nutrition literacy as well as for the number of different foods consumed by the child are tested but excluded due to high collinearity with SES.

Model performance is evaluated by comparing the average Root Mean Squared Error (RMSE) of 1,000 out-of-sample predictions. We use cross-validation with a training and test set size of 50% each. Table S3 shows the results of in- and out-of-sample predictions.

Table S3: Comparing model performance

|  | Potential buyer | |  | Current buyer | |
| --- | --- | --- | --- | --- | --- |
|  | Avg. RMSE  in-sample | Avg. RMSE out-of-sample |  | Avg. RMSE  in-sample | Avg. RMSE out-of-sample |
| Baseline Model (FE) | 149.35 | 150.76 |  | 706.80 | 706.89 |
| Tobit Model | 176.61 | 178.44 |  | 706.87 | 706.96 |
| Hurdle Model | 153.95 | 155.80 |  | 707.78 | 707.59 |
| Heckman Model | 157.85 | 159.67 |  | 706.87 | 706.95 |

Source: Authors’ calculation. Note that demand of current buyers is modelled on a logarithmic scale. Since the RMSE is a scale-dependent measure, the RMSE’s are comparable between different models but not between current and potential buyers.

The baseline model performs best both for potential and current buyers. The small difference between in- and out-of-sample RMSE further indicates fairly well specified models. As expected, the model performances differ much less for current buyers than for potential buyers, which is what we expect as bounding is less of an issue among current buyers.

1. Illustrative examples of cost-effectiveness calculations

The two examples in Fig. S3 illustrate the cost-effectiveness calculations:

- In case (A) a hypothetical price subsidy of 6m USD reduces production losses by 4m USD and averts 40,000 DALYs. The resulting net social costs amount to 2m USD and the price per DALY averted is 50 USD/DALY (net social costs of 2m / 40,000 DALYs).
- In case (B) the intervention leads to a stronger reduction in production losses of 7m USD resulting in a negative social cost of −3m USD. The price per DALY averted is negative at −75 USD/DALY. The intervention is therefore cost-saving, as it not only averts DALYs but also saves net social costs. The cost-effectiveness of the intervention would be different if the provider of the subsidy does not take into account the productions losses averted. In this case the cost-effectiveness ratio is calculated by dividing the cost of the price subsidies by the number of DALYs averted, obtaining a cost of 150 USD/DALY for both case (A) and case (B).

Fig. S3: Illustration of cost-effectiveness calculation

| (A) | (B) |
| --- | --- |
| 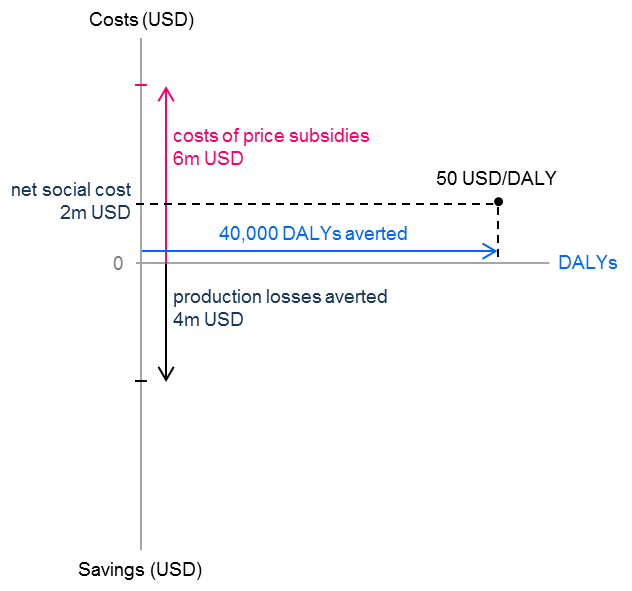 | 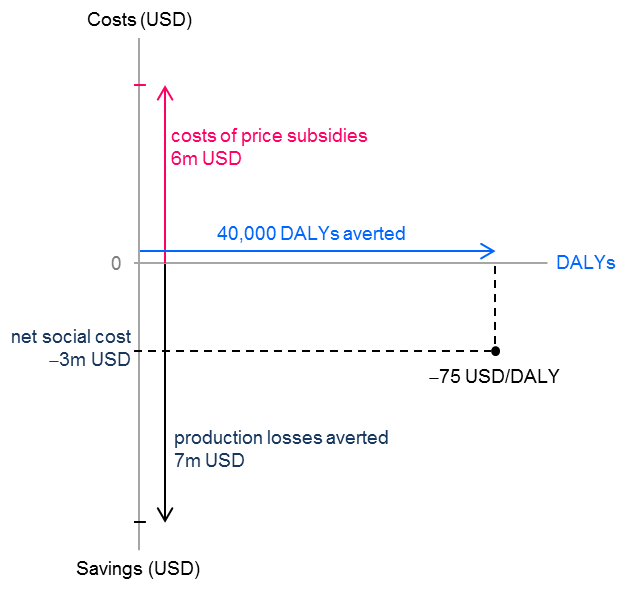 |

1. Results of the market survey

Table S4 shows the results of the hypothetical marketing experiment after applying the ex-post correction for hypothetical questions.

Table S4: Marketing experiment, descriptive overview

|  | Current buyers | | | Current non-buyers | | | |
| --- | --- | --- | --- | --- | --- | --- | --- |
|  | offered usually bought brand | | | offered 25 g de-branded packs | | | |
|  | at discounted price | | | at discounted price | | | |
| Discount | N  of offers | Mean current demand | Mean  hypothetical ∆ demand | N  of 1st. offers | N  accepting 1st.offer | Mean  current demand | Mean  hypothetical ∆ demand |
|  |  |  |  |  |  |  |  |
| 20% | 256 | 197 | 147 | 234 | 71 | 0 | 219 |
|  |  | (13) | (24) |  |  |  | (15) |
| 40% | 204 | 186 | 177 | 247 | 77 | 0 | 206 |
|  |  | (12) | (33) |  |  |  | (13) |
| 60% | 100 | 167 | 437 | 247 | 24 | 0 | 216 |
|  |  | (13) | (112) |  |  |  | (28) |
| 80% | 50 | 170 | 516 | 196 | 12 | 0 | 227 |
|  |  | (19) | (138) |  |  |  | (44) |
| Total | 610 | 186 | 235 | 924 | 184 | 0 | 214 |

All observations were weighted according to district size. Columns 4 and 8 show the average change of demand in grams per week induced by the discount on the currently purchased product (current buyers) or the unbranded product (potential buyers) (standard errors in parentheses).

Fig. S4 illustrates that the consumption of fortified formulas decreases by 72% between the age of 6 and 23 months.

Fig. S4: Consumption of FPCF by age


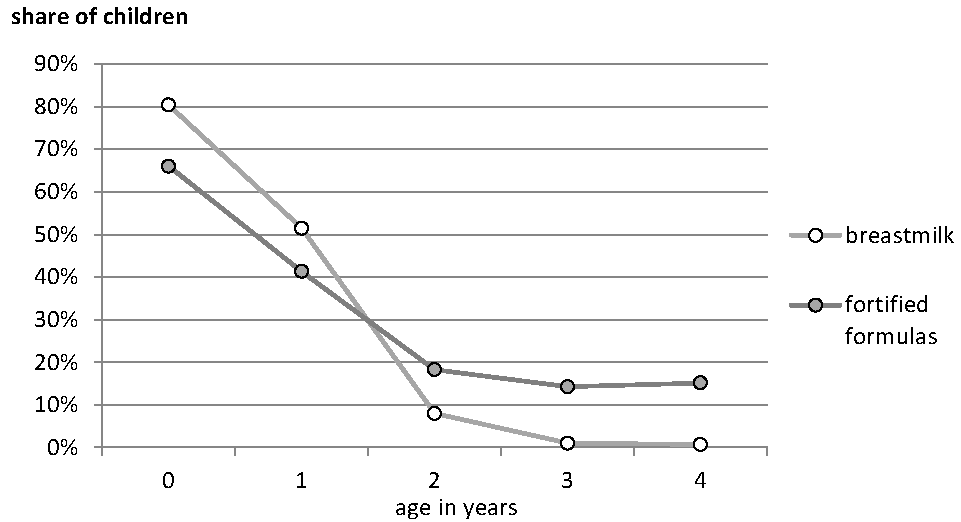


Age zero includes 6-11-month old children. Fortified formulas include packaged infant cereals and powdered milk.

Source: Authors’ calculation.

1. Decomposition of effect of subsidies

Fig. S5: Decomposition of demand and cost effects of subsidies


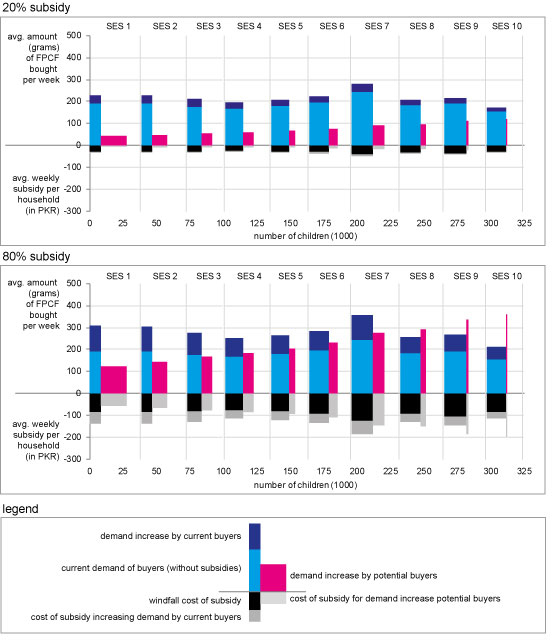


The horizontal axis shows the different numbers of 6-17-month-old children per SES. The upper panel illustrates the average amount of FPCF bought per week by current buyers of FPCF, potential buyers and non-buyers.

The windfall cost corresponds to the cost of the price subsidies on the FPCF that current buyers would have been bought even in absence of the subsidy. This windfall cost increases substantially as the subsidies increase.

Source: Authors’ calculation.

1. Sensitivity analyses

In order to test the robustness of our results we conducted an univariate and a probabilistic sensitivity analysis. For ease of interpretation both sensitivity analyses were conducted for only a single scenario: 50% discount for the poorest 60% of the population. The base case values of this scenario are intervention costs of 7.5m USD, 9,722 DALYs averted and a net saving of 498 USD per DALY averted.

Probabilistic sensitivity analysis

With a probabilistic sensitivity analysis (PSA) we estimated the distribution of our main. These distributions are comparable to the cost-effectiveness acceptability curves usually shown in cost-effectiveness analysis.

We randomly varied all model parameters within predefined distributions and then ran the model 1000 times. Table S5 reports the types of distribution assumed for the different categories of model parameters. Where information on the variability of the parameters was not available from the literature, we chose a SD of 20% of the mean. Where variables differed across SES (marginal effects and intervention costs) we accounted for the interdependence of values between SES. It is implausible for a single random draw to combine very low marginal effects in one SES and very high marginal effects in another SES. We therefore drew random quantiles and then looked up the corresponding value in the distribution with SES specific parameters. Thus, within a single random draw all SES will always have a marginal effect on the same quantile of their individual distribution.

Table S5: Assumptions on distributions of model parameters

| **Parameter** | **Distribution** |
| --- | --- |
| Unrestricted parameters, such as cognitive score difference due to IDA | Normal |
| Non-negative parameters such as duration of illnesses and risk estimates | Lognormal |
| Parameters with 0,1 range, such as disability weights and share of IDA | Beta |
| Cost parameters, Marginal effects, Incidence rate | Gamma |

The following Figures show the distribution of intervention costs, DALYs saved and cost per DALY for a 50% discount for the poorest 60% of the population. The median simulated value is comparable with the base case for all three measures. Fig. S6 shows the distribution of intervention costs. They vary roughly between 6.5 and 8.6 in 95% of all simulated cases.

Fig. S6: Distribution of cost of intervention

Source: Authors’ calculation.

The variation of the results is within the expected limits also for the number of DALYs averted ranging roughly between -35% and +35% compared to the median in 95% of all simulations. In particular, in 95% of all simulations the number of DALYs averted lies between 6,407 and 13,009 DALYs (Fig. S7).

Fig. S7: Distribution of DALYs averted

Source: Authors’ calculation.

Fig. S8 shows the distribution of the net cost, respectively the net saving, per DALY averted. In 95% of the simulated cases, the result lies between a net cost of 434 USD and a net saving of 2,450 USD per DALY averted. The result changes qualitatively from a net saving to a net cost at the upper quartile of model runs (at the upper 25% of cases).

Fig. S8: Distribution of net cost per DALY

Source: Authors’ calculation.

Overall we found that variation in the model results is considerable. This is not surprising given the complexity of the question and the many data sources combined in our model. The probabilistic sensitivity analysis shows however that the uncertainty is limited.

Univariate sensitivity analysis regarding the spot UIC values

Due to day-to-day within-person variation of iodine intake, we might overestimate the prevalence of IoD by using spot UIC values ^(14, 15)^. We, therefore, conducted a univariate sensitivity analysis, in which we adjusted the spot UIC distribution with a correcting factor. This correcting factor was calculated as the difference between the unadjusted and adjusted (to within-person variability) median iodine intake obtained from Zimmermann et al. ^(14)^. We *first* calculated the average adjusted median iodine intake, weighted by the number of children in each age-group, for each country, as provided in Table 4 in Zimmermann et al. ^(14)^. *Second*, we compared this weighted average of the adjusted median iodine intake to the unadjusted median of iodine intake provided in Table 2 for each examined country and calculated the average weighted by the sample size of each country. *Third*, we translated this difference from iodine intake to UIC value based on equation [3] (or (1) in Zimmermann et al. ^(14)^). *Fourth*, we added this difference to our initial UIC distribution and re-ran our model. For ease of interpretation this was conducted only for a single scenario. Table S6 shows the percentage change of the prevalence of IoD and of the main three results when adding the correcting factor to the initial UIC distribution.

Table S6: Prevalence of IoD and cost-effectiveness results before and after adjusting the UIC distribution with the correcting factor

|  | **Mild IoD** | **Moderate IoD** | **Severe IoD** | **Cost of intervention  (m USD)** | **Production losses averted  (m USD)** | **DALYs averted** | **Net cost per DALY averted**  **(USD)** |
| --- | --- | --- | --- | --- | --- | --- | --- |
| **Baseline** | 26% | 11% | 2% | 7.5 | 12.4 | 9,722 | -498 |
| **Adjusted** | 22% | 2% | 0% | 7.5 | 7.9 | 7,491 | -50 |
| **%- change** | -15% | -82% | -100% | 0% | -36% | -23% | -90% |

Source: Authors’ calculation.

Correcting for the within-person variance of iodine intake leads to a 90% smaller cost saving ratio per DALY averted. Due to a smaller prevalence of IoD the production losses and DALYs averted fall by 36% and 23% respectively, while the intervention costs remain the same.

References

1. Stoltzfus RJ, Mullany l, Black RE (2004) Iron deficiency anemia. In: *Comparative quantification of health risks: the global and regional burden of disease due to 25 selected major risk factors.* pp. 163-209. [Ezzati M, editor]. Cambridge: Harvard University Press.

2. Wieser S, Brunner B, Tzogiou C et al. (2017) Societal Costs of Micronutrient Deficiencies in 6- to 59-month-old Children in Pakistan. *Food Nutr Bull*. Published online: 2 October 2017. doi: 10.1177/0379572117720012.

3. Zimmermann M, Andersson M (2014) Development of a new framework to estimate the global burden of disease due to iodine deficiency. In: *Methodological approaches to estimating global and regional prevalences of vitamin and mineral deficiencies.* Report on the Joint World Health Organization/US Centers for Disease Control and Prevention Technical Consultation. Atlanta, USA, 7–9 December 2010. pp. 65–77. Geneva: WHO.

4. Kassebaum NJ, Jasrasaria R, Naghavi M et al. (2014) A systematic analysis of global anemia burden from 1990 to 2010. *Blood* **123**, 615-624. doi: 10.1182/blood-2013-06-508325.

5. Rastogi T, Mathers C. Global burden of iron deficiency anemia in the year 2000. Geneva: World Health Organization; 2002.

6. Lozoff B, Jimenez E, Smith JB (2006) Double burden of iron deficiency in infancy and low socioeconomic status. A longitudinal analysis of cognitive test scores to age 19 years. *Arch Pediatr Adolesc Med* **160**, 1108-1113. doi: 10.1001/archpedi.160.11.1108.

7. Stein AJ, Meenakshi JV, Qaim M et al. (2005) *Analyzing the health benefits of biofortified staple crops by means of the disability-adjusted life years approach: a handbook focusing on iron, zinc and vitamin A.* Series. Washington, DC and Cali: HarvestPlus Technical Monograph 4.

8. Salomon JA, Haagsma JA, Davis A et al. (2015) Disability weights for the Global Burden of Disease 2013 study. *Lancet Glob Health* **3**, e712-e723. doi: 10.1016/S2214-109X(15)00069-8.

9. Brabin BJ, Premji Z, Verhoeff F (2001) An analysis of anemia and child mortality. *J Nutr* **131**, 636S-648S.

10. Imdad A, Herzer K, Mayo-Wilson E et al. (2010) Vitamin A supplementation for preventing morbidity and mortality in children from 6 months to 5 years of age. *Cochrane Database Syst Rev* CD008524. doi: 10.1002/14651858.CD008524.pub2.

11. Horton S, Ross J (2003) The economics of iron deficiency. *Food Pol* **28**, 51-75. doi: 10.1016/s0306-9192(02)00070-2.

12. Blumenschein K, Blomquist GC, Johannesson M et al. (2008) Eliciting willingness to pay without bias: evidence from a field experiment. *Economic Journal* **118 (January)**, 114-137.

13. Whitehead JC, Cherry TL (2007) Willingness to pay for a green energy program: a comparison of ex-ante and ex-post hypothetical bias mitigation approaches. *Resour Energy Econ* **29**, 247-261.

14. Zimmermann MB, Hussein I, Al Ghannami S et al. (2016) Estimation of the Prevalence of Inadequate and Excessive Iodine Intakes in School-Age Children from the Adjusted Distribution of Urinary Iodine Concentrations from Population Surveys. *J Nutr* **146**, 1204-1211.

15. Carriquiry AL (2003) Estimation of usual intake distributions of nutrients and foods. *The Journal of Nutrition* **133**, 601S-608S.
